# Downregulation of long noncoding RNA HCP5/miR-216a-5p/ZEB1 axis inhibits the malignant biological function of laryngeal squamous cell carcinoma cells

**DOI:** 10.3389/fimmu.2022.1022677

**Published:** 2022-09-30

**Authors:** Sen Zhang, Hui Huangfu, Qinli Zhao, Yujun Li, Lina Wu

**Affiliations:** ^1^ Department of Otolaryngology Head and Neck Surgery, The First Hospital, Shanxi Medical University, Taiyuan, China; ^2^ Shanxi Key Laboratory of Otorhinolaryngology Head and Neck Cancer, Shanxi Medical University, Taiyuan, China; ^3^ Department of Pathology, The First Hospital, Shanxi Medical University, Taiyuan, China

**Keywords:** invasion, lncRNA, miRNA, migration, proliferation, ZEB1

## Abstract

Previous studies find that long noncoding RNA human leukocyte antigen complex P5 (HCP5) is regarded as an oncogene *via* accelerating cancer cell growth, invasion, metastasis, vascularization, and drug resistance in renal cell carcinoma, gastric cancer, and colorectal cancer. Nevertheless, the effect and regulatory mechanism of HCP5 in laryngeal squamous cell carcinoma (LSCC) remains unknown. In this study, HCP5 expression levels were confirmed to be prominently raised in LSCC cell lines. HCP5 knockdown reduced cell proliferation and migration and invasive ability of LSCC cell lines. Furthermore, miR-216a-5p was confirmed to sponge HCP5, and its expression was prominently downregulated in LSCC cell lines and upregulated in HCP5-silenced LSCC cell lines. miR-216a-5p overexpression downregulated the cell proliferation and migration and invasive ability of LSCC cells. Additionally, the protein level of zinc finger E-box binding homeobox 1 (ZEB1), one target gene of miR-216a-5p, was highly expressed in LSCC cell lines, and its expression level was downregulated by HCP5 knockdown and miR-216a-5p overexpression. An miR-216a-5p inhibitor reversed the effect of HCP5 knockdown on the proliferation and migration and invasive ability of LSCC cells. In conclusion, knocking down HCP5 may be a strategy to suppress the malignant biological function *via* regulating miR-216a-5p/ZEB1. Therefore, HCP5 may become a prospective therapeutic target for LSCC.

## Introduction

Head and neck cell carcinoma is an invasive malignant tumor that includes oral, hypopharyngeal, and laryngeal cancer, and its incidence ranks sixth among various types of tumors (1). In particular, laryngeal squamous cell carcinoma (LSCC) is the usual cancer type of the larynx, accounting for approximately 90% of all laryngeal carcinomas (2). Surgical excision is an effective treatment method for early LSCC, but this strategy is limited for advanced LSCC (3). Hence, the identification of potential targets involved in the occurrence and metastasis contributes to exploring novel targets for treatment of LSCC.

Long noncoding RNA (lncRNA) has been considered in previous studies to play a key role in the progression, vascularization, and aggressive behavior of cancer (4, 5). In LSCC, dysregulated lncRNA, such as SNHG16, PTCSC3, and XIST, can regulate LSCC cell growth, metastasis, angiogenesis, and chemoresistance (6–8). Human leukocyte antigen complex P5 (HCP5), an lncRNA, is located on human chromosome 6p21.33 (9). Previous studies find that HCP5 is regarded as an oncogene *via* accelerating cancer cell growth, metastasis, and drug resistance in renal cell carcinoma, gastric cancer, and colorectal cancer (10–12). Additionally, HCP5 expression is confirmed to be prominently increased in oral SCC, which has a key role in promoting cancer cell invasion (13). These studies suggest that HCP5 is an oncogene. Nevertheless, the biological behavior and regulatory mechanism of HCP5 in LSCC remains unknown. At the same time, oral SCC and LSCC belong to head and neck cell carcinoma; hence, we infer that HCP5 also plays an oncogenic role in LSCC. Therefore, this study selected HCP5 to study its function in LSCC and to clarify whether it could be a potential therapeutic target for LSCC.

Therefore, we determined HCP5 expression and roles in LSCC cell lines. We also investigated the regulatory mechanism of HCP5 in LSCC by sponging microRNAs (miRNAs).

## Methods

### Cell culture and transfection

Human keratinocytes HaCaT and LSCC cell including Tu-686, SNU899, SNU46, Tu-177, and AMC-HN-8 (ATCC, Manassas, VA, USA) were cultured as previously described (14). Negative control miRNA mimic/inhibitor (NC mimic and NC inhibitor), miR-216a-5p mimic/inhibitor, small interference RNAs (siRNAs) targeting RNA sequence of HCP5, and negative control siRNA were synthesized from RiboBio (Guangzhou, China). Each of the products (50 nM) was transfected with Lipofectamine 3000 (Life technologies, Carlsbad, CA, USA). Their sequences are shown as follows: 5′‐GGCAGATTACAATTACAATCAAGDTDT‐3′ (si-HCP5-1), 5′‐GAGATGT CTTTGATTTTTAAAATDTDT‐3′ (si-HCP5-2), and 5′‐ATGATGTTGTCAATGAAATAAAGDTDT‐3′ (si-HCP5-3). The sequence of negative control siRNA was 5′‐TTCTCCGAACGTGTCACGTDTDT‐3′.

### Reverse transcription quantitative PCR (RT-qPCR)

HaCaT and LSCC cell lines and transfected Tu-177 and Tu-686 cells were washed with PBS, and then, 1 ml TRIzol reagent (Invitrogen) was used in each well to isolate total RNA. To analyze the expression level of HCP5, a reverse transcription reaction to obtain cDNA was carried out according to the method of the PrimeScript™ RT reagent Kit (TaKaRa, Dalian) using reverse transcription primer olig dT. To analyze miR-216a-5p expression levels, a reverse transcription reaction to obtain cDNA was carried out according to the method of the HyperScript III miRNA 1st Strand cDNA Synthesis Kit (by stem-loop) (NovaBio, Shanghai, China). The QPCR reaction system (20 μl) was prepared according to the instructions of SYBR GREEN qPCR Super Mix (Invitrogen). PCR reaction was performed using the ABI 7500 Real-time PCR system (Applied Biosystems, Foster City, CA, USA). GAPDH and U6 were analyzed as the internal control gene for HCP5 and miR-216a-5p, respectively. The 2^−ΔΔct^ method was used to calculate the relative expression level of HCP5 and miR-216a-5p (15). Primers (5′‐3′) for HCP5 are GACTCTCCTACTGGTGCTTGGT (forward primer, F) and CACTGCCTGGTGAGCCTGTT (reverse primer, R); Primers (5′‐3′) for GAPDH are GCTCATTTGCAGGGGGGAG (F) and GTTGGTGGTGCAGGAGGCA (R). Primers (5′‐3′) for miR-216a-5p are ACACTCCAGCTGGGAAGGGTAATCTCAGCTGGCAA (F) and CTCAACTGGTGTCGTGGA (R). Primers (5′‐3′) for U6 are CTCGCTTCGGCAGCACA (F) and AACGCTTCACGAATTTGCGT (R).

### Assessment of cell proliferation

Twenty-four hours after transfection, 1×10^4^ transfected Tu-177 and Tu-686 cells were seeded in 96-well plates. After culture for 0, 24, 48, and 72 h, 10 μl AQueous One Solution reagent (Promega) was added into each well. After cultivation for 4 h, the optical density at an absorbance of 490 nm (OD_490 nm_) was measured.

### Transwell migration/invasion assay

To assess the migrated ability, 1×10^5^ transfected Tu-177 and Tu-686 cells of each group (in serum-free culture medium) were seeded in the upper Transwell chamber (Corning, Corning, NY, USA), and 600 µl culture medium supplemented with 10% serum was put into the lower well. After culture for 24 h, cells on the lower surface of the membrane were stained with crystal violet solution. Photos (100×) were taken, and cells in each photo were counted. For the invasion assay, the upper Transwell chamber was precoated with Matrigel (BD Biosciences, Bedford, MA, USA), and the remaining steps are the same as the migration operation.

### Dual‐luciferase reporter gene assay

StarBase 2.0 (16) was used to predict the possible sponged miRNAs of HCP5. TargetScan version 7.1 and StarBase version 2.0 were used to predict the potential target genes of miR-216a-5p. The wild-type HCP5 and ZEB1 3′-UTR (WT-HCP5 and WT-ZEB1) or mutant HCP5 and ZEB1 3′-UTR (Mut-HCP5 and Mut-ZEB1) were cloned into a psi-CHECK2 vector. Thirty nanograms of either WT-HCP5 and Mut-HCP5 or WT-ZEB1 and Mut-ZEB1 were cotransfected with 50 nM of either miR-216a-5p mimics or NC mimic. After 48 h, Renilla and firefly luciferase activity were measured according to the instructions of the Dual‐Luciferase Assay kit (Promega), and their ratio (Renilla/firefly) was used as the relative luciferase activity to evaluate whether miR-216a-5p has binding sites on the predicted sequence of HCP5 and ZEB1 3′-UTR.

### Western blot analysis

The total protein (30 μg per lane) was isolated using RIPA buffer. After 10% SDS-PAGE, proteins were transferred onto methanol-pretreated polyvinylidene fluoride membranes. Following this, membrane blocking, primary antibody incubation, and secondary antibody incubation were performed according to conventional methods. The dilution of zinc finger E-box binding homeobox 1 (ZEB1) monoclonal antibody (14-9741-80, Ebioscience, San Diego, CA, USA) and loading control GAPDH monoclonal antibody (MA5-15738, Ebioscience) were 1:1000 and 1:5000, respectively. The dilution of horseradish peroxidase-conjugated secondary antibody goat anti-mouse IgG (G-21040, Ebioscience) was 1:1000. Enhanced chemiluminescent reagent (Thermo Scientific Pierce, Rockford, IL, USA) was used to visualize the protein abundance in the membrane.

### Statistical analysis

The GEPIA website was used to analyze HCP5 expression in 44 normal and 519 head and neck squamous cell carcinoma tissues. One-way analysis of variance (ANOVA) followed by Dunnett’s test were used to analyze to statistical difference of all the experimental data by SPSS 19.0 software (SPSS Inc., Chicago, IL, USA). All data in the bar graphs are presented as mean ± standard deviation. *p <.05 was considered statistically significant.

## Results

### HCP5 was upregulated in LSCC cell lines

**Figure 1:**
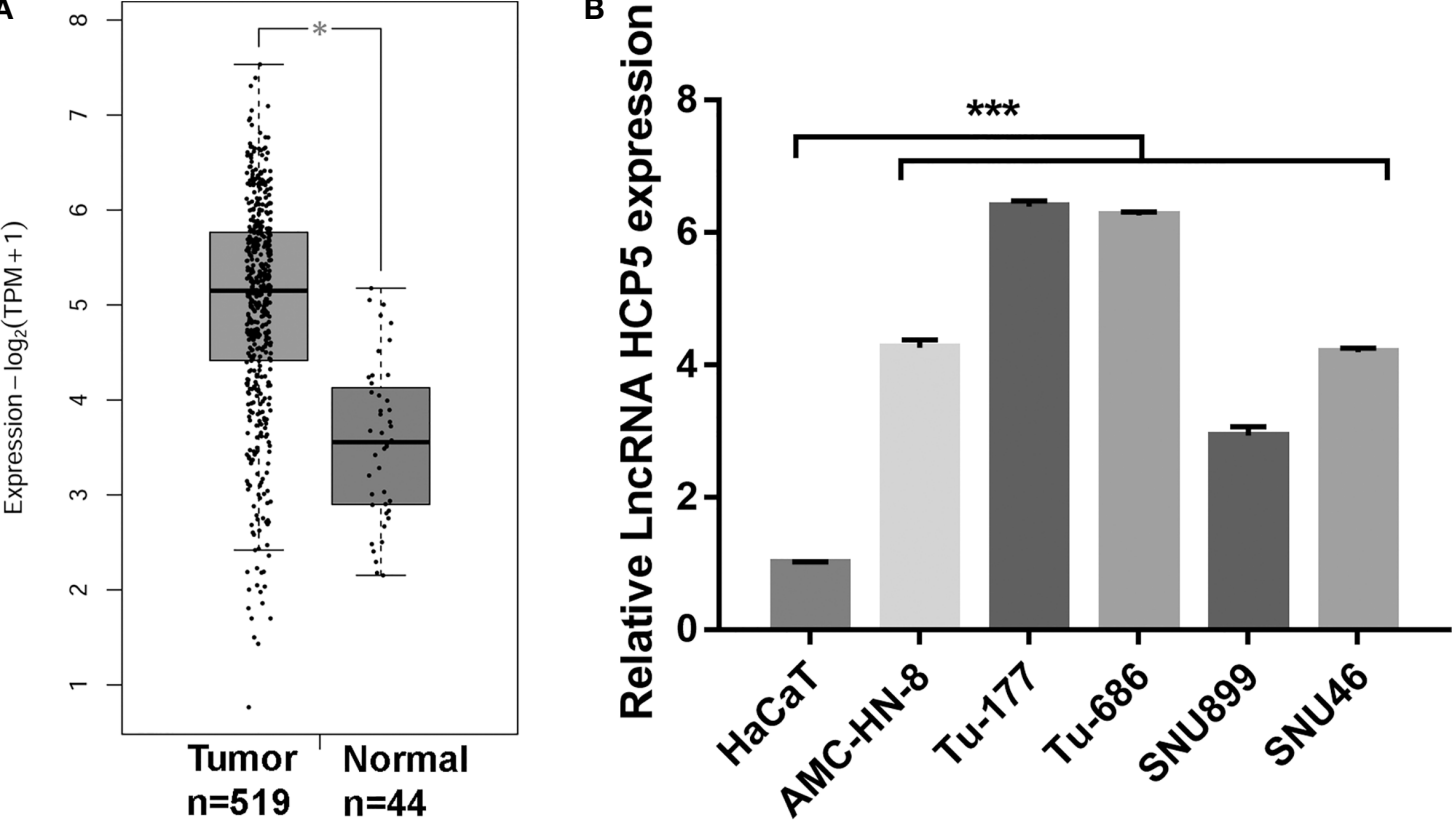
HCP5 expression was prominently raised in head and neck squamous cell carcinoma tissues and LSCC cell lines. **(A)** HCP5 expression in normal and tumor tissues was analyzed by GEPIA *P < 0.05. **(B)** HCP5 expression was measured by RT-qPCR in LSCC cell lines including Tu-177, Tu-686, SNU899, AMC-HN-8, and SNU46 and normal keratinocytes HaCaT. *n*=3, ***p <.001, vs HaCaT.

To understand the HPC5 expression in LSCC tissues and cells, HCP5 expression in tumor tissues and LSCC cells were measured by GEPIA and RT-qPCR, respectively. HCP5 expression in tumor tissues was prominently higher than that in the normal group **(** **Figure 1A**). HCP5 expression was prominently raised in all LSCC cells, particularly in Tu-177 and Tu-686, compared with the expression in HaCaT cells (**Figure 1B**). Thus, we chose the Tu-177 and Tu-686 cell lines for further experiments.

### Silenced HCP5 suppressed the proliferation and metastasis in Tu-177 and Tu-686

**Figure 2:**
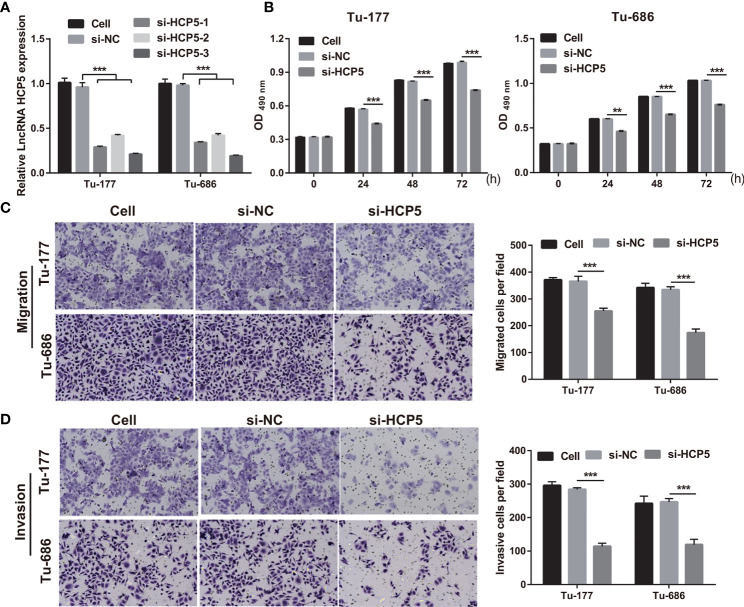
Silenced HCP5 inhibited biological behaviors of Tu-177 and Tu-686. **(A)** HCP5 expression was quantified by RT-qPCR in Tu-177 and Tu-686 after transfection with si-HCP5 or si-NC. **(B)** The effect of silenced HCP5 on proliferation in Tu-177 and Tu-686 cells were determined by MTS assay. **(C, D)** The impact of silenced HCP5 on the migration and invasion of Tu-177 and Tu-686 were tested by Transwell assay (100 × magnification). *n*=3, **p < 0.01 and ***p < .001.

To study the function of HCP5 in LSCC, HCP5 was silenced by transfecting si-HCP5 (si-HCP5-1/2/3) into both Tu-177 and Tu-686 cells. RT-qPCR results shows that si-HCP5 transfection significantly downregulated the HCP5 expression in both Tu-177 and Tu-686 cells, especially for si-HCP5-3 (**Figure 2A**). Thus, we chose si-HCP5-3 for further experiments. Next, silenced HCP5 (the si- HCP5 group) significantly reduced the proliferation, migration, and invasion abilities of Tu-177 and Tu-686 compared with the si-NC group (**Figures 2B–D**).

### HCP5 served as a sponge for miR-216a-5p

**Figure 3:**
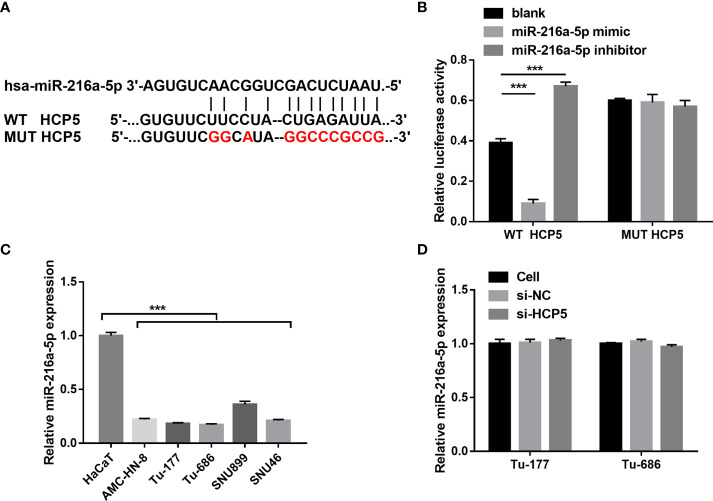
HCP5 served as a sponge for miR-216a-5p. **(A)** Bioinformatic analysis predicted the binding sites between HCP5 and miR-216a-5p. **(B)** A dual-luciferase reporter assay indicated that HCP5 directly bound to miR-216a-5p. **(C)** miR-216a-5p expression in LSCC cell lines and normal keratinocytes HaCaT was measured by RT-qPCR. **(D)** Effect of silenced HCP5 on the miR-216a-5p expression in LSCC cell was measured by RT-qPCR. *n*=3, ***p <.001.

To characterize the downstream mechanisms underlying the inhibitory effect of HCP5 in LSCC cells, the sponged miRNAs of HCP5 were predicted using StarBase 2.0 databases. Among miRNAs, miR-216a-5p was found to be the possible sponged-miRNA of HCP5 (**Figure 3A**). An miR-216a-5p mimic prominently lessened the relative luciferase activity in the WT-HCP5 group while not affecting that in the mut-HCP5 group (**Figure 3B**). These results indicate a direct bond between HCP5 and miR-216a-5p. miR-216a-5p expression was prominently lower in LSCC cells than that in HacaT cells, which was not regulated by HCP5 knockdown (**Figures 3C, D**). All results found that HCP5 only sponged miR-216a-5p in LSCC cells.

### Overexpression of miR-216a-5p has inhibitory effects on the proliferation and metastasis in Tu-177 and Tu-686

**Figure 4:**
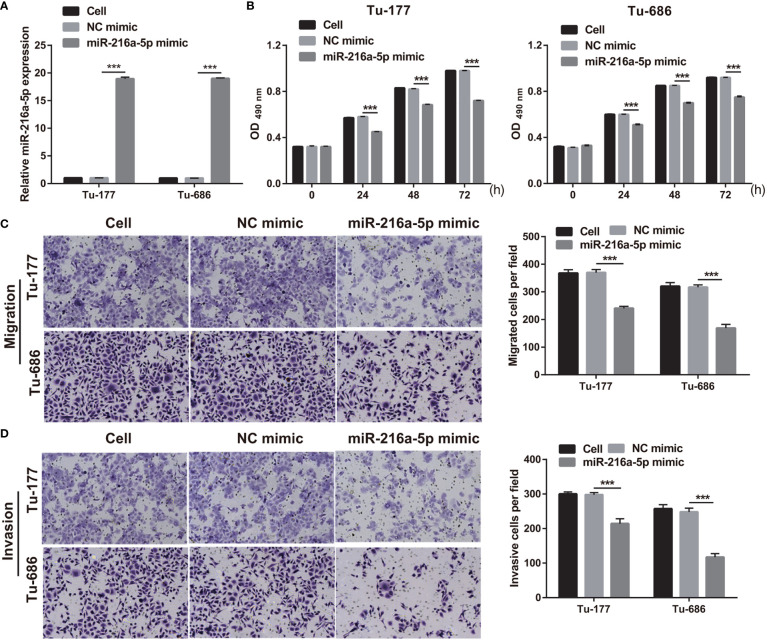
Overexpression of miR-216a-5p suppressed LSCC biological behaviors. **(A)** miR-216a-5p expression in Tu-177 and Tu-686 after transfection was quantified. **(B)** Effect of overexpression miR-216a-5p on proliferation in Tu-177 and Tu-686 were determined by MTS assay. **(C, D)** Effect of overexpression miR-216a-5p on the migration and invasion in Tu-177 and Tu-686 were assessed by Transwell assay (100 × magnification). *n*=3, ***p <.001.

To study the function of miR-216a-5p in LSCC, miR-216a-5p mimic was transfected into Tu-177 and Tu-686 and it was found that miR-216a-5p expression was prominently improved in LSCC cells (**Figure 4A**). The proliferation, migration, and invasion abilities of Tu-177 and Tu-686 in the miR-216a-5p mimic group were prominently lower than that in the NC mimic group (**Figures 4B–D**).

### Silenced miR-216a-5p can attenuate the effect of si-HCP5 in Tu-177 and Tu-686

**Figure 5:**
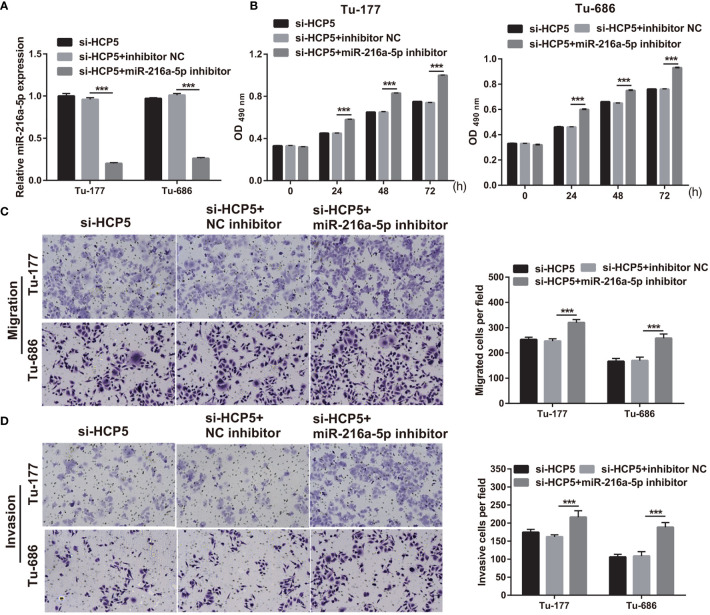
miR-216a-5p-silenced can attenuate si-HCP5 effect in LSCC cells. **(A)** miR-216a-5p expression was quantified by qRT-PCR in Tu-177 and Tu-686 cells after cotransfected si-HCP5 and miR-216a-5p inhibitor. **(B)** The effect of cotransfected si-HCP5 and miR-216a-5p inhibitor on proliferation in Tu-177 and Tu-686 were determined by MTS assay. **(C, D)** The effect of cotransfected si-HCP5 and miR-216a-5p inhibitor on the migration and invasion of Tu-177 and Tu-686 were assessed by Transwell assay; the presented pictures were captured with 100× magnification. *n*=3, ***p <.001.

To further verify the correlation between miR-216a-5p and HCP5 in LSCC, si-HCP5 and miR-216a-5p inhibitors were cotransfected into Tu-177 and Tu-686. miR-216a-5p expression was prominently inhibited after cotransfection (**Figure 5A**). The proliferation, migration, and invasion abilities in the si-HCP5+miR-216a-5p inhibitor group were prominently enhanced compared with those in the si-HCP5+NC inhibitor group (**Figures 5B–D**).

### ZEB1 is a regulated target gene for miR-216a-5p

**Figure 6:**
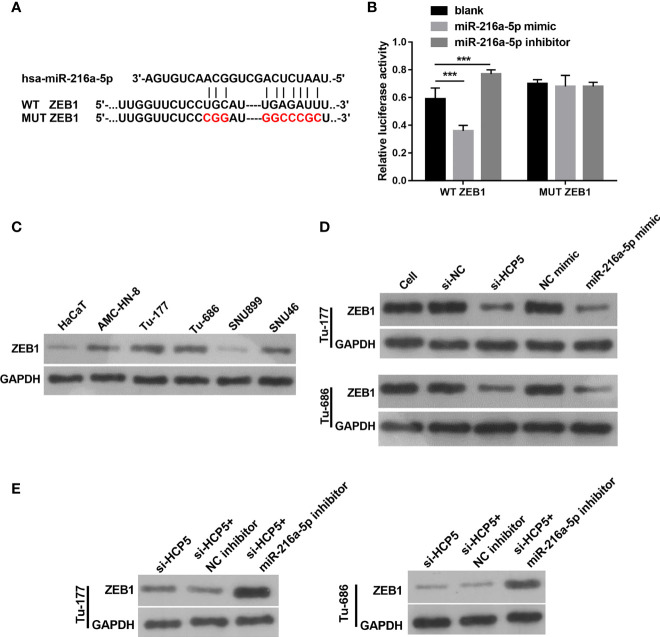
ZEB1 is a directly regulated target gene of miR-216a-5p. **(A)** The direct binding sites between ZEB1 3′-UTR and miR-216a-5p were predicted by bioinformatic analysis. The mutant binding site of ZEB1 was also exhibited. **(B)** A dual-luciferase reporter assay certified that miR-216a-5p directly binds to ZEB1. **(C)** Protein levels of ZEB1 in LSCC cell lines was measured by Western blot, respectively. **(D)** The effect of silenced HCP5 or overexpression of miR-216a-5p on the protein expression of ZEB1 in Tu-177and Tu-686 cells was measured by Western blot. **(E)** The effect of cotransfected si-HCP5 and miR-216a-5p inhibitor on ZEB1 protein in LSCC cells was quantified by Western blot. *n*=3, ***p <.001.

The target site for miR-216a-5p binding was found to be the 3′-UTR of ZEB1 (**Figure 6A**). An miR-216a-5p mimic significantly decreased the relative luciferase activity in the WT-3′-UTR ZEB1 group, but did not affect MUT-3′-UTR ZEB1 (**Figure 6B**), which indicates the direct binding between miR-216a-5p with the 3′-UTR of ZEB1. The protein level of ZEB1 was higher in LSCC cells than in HacaT cells (**Figure 6C**). HCP5 silenced or miR-216a-5p overexpression significantly decreased ZEB1 protein in both Tu-177and Tu-686 cells (**Figure 6D**). ZEB1 protein was obviously enhanced 48 h after cotransfection of si-HCP5 and miR-216a-5p inhibitor in both Tu-177and Tu-686 cells (**Figure 6E**).

## Discussion

Tumor metastasis is an important reason for poor prognoses in LSCC patients. Here, we elucidated that HCP5 is prominently upregulated in LSCC cell line, and HCP5 downregulation inhibited proliferation, migration, and invasion, suggesting that HCP5 is an oncogene in LSCC. miR-216a-5p was sponged by HCP5 in LSCC. miR-216a-5p overexpression reduced proliferation, migration, and invasion in LSCC, suggesting that it is a tumor suppressor miRNA. Furthermore, silenced miR-216a-5p reversed the function of HCP5 silencing, suggesting that HCP5 promotes LSCC progression *via* sponging miR-216a-5p. Moreover, ZEB1 is a regulated target gene for miR-216a-5p. Silencing its expression reversed the effect of HCP5 silencing on ZEB1 expression, suggesting that HCP5 enhanced ZEB1 protein by sponging miR-216a-5p. These results suggest that HCP5 promotes LSCC progression by inhibiting the miR-216a-5p/ZEB1 axis. This study is the first to discover the role and mechanism of HCP5 in LSCC, which enriches the theory of the occurrence and development mechanism of LSCC.

HCP5 closely contributes to tumor initiation and progression. HCP5 is a novelty diagnostic and prognostic biomarker in gastric and bladder cancers (17, 18). In addition, HCP overexpression enhanced chemoresistance in gastric cancer and esophageal carcinoma (19, 20). Besides this, HCP5 was prominently upregulated and acted as an oncogene in pancreatic cancer, bladder cancer, gastric cancer, and cutaneous squamous cell carcinoma (21–24). Consistent with previous reports of other cancers, HCP5 expression was also upregulated in LSCC cells, and HCP5 was an oncogene.

In recent years, increasing evidence has found that abnormal expression and dysfunction of miRNAs also plays important roles in LSCC (25, 26). Here, miR-216a-5p was downexpressed in LSCC cells and acts as an anticancer miRNA in the LSCC. miR-216a-5p expression was elucidated to prominently reduce in breast, pancreatic, colorectal, and small cell lung cancers and reversing its expression significantly suppressed cancer development (27–31). Importantly, miR-216a-5p expression was prominently reduced in esophageal SCC and promotes its indeterminate growth (32). Studies have found that miR-216a-5p acts as an anticancer miRNA, which is consistent with its role in LSCC.

Besides this, lncRNA is elucidated to enhance the expression of targeted genes by sponging with miRNAs (33). HCP5 promotes cancer development by sponging miR-140-5p, miR−138−5p, miR-29b-3p, and miR-143-3p (21–24). In addition, HCP5 can sponge miR-216a-5p in cervical cancer (34). Here, we confirm that HCP5 promotes LSCC progression *via* sponging miR-216a-5p.

Next, ZEB1 is a directly regulated target gene for miR-216a-5p. ZEB1 expression was prominently upregulated in NSCLC, which can judge the overall survival rate (35). ZEB1 accumulation enhanced the invasion and EMT of liver and gastric cancer cells (36, 37). Importantly, ZEB1 acts as an oncogene and is closely related to EMT and prognosis in LSCC (38, 39). Our study shows that ZEB1 expression was enhanced in LSCC cells, and it was inhibited by silencing HCP5 and promoted by cosilencing HCP5 and miR-216a-5p. These results reveal that ZEB1 is a downstream targeted gene that is regulated by the HCP5/miR-216a-5p axis. Previous conclusions show that lncRNA-SNHG16 can regulate miR-216a-5p/ZEB1 and promote tumor development in cervical cancer tissues, which partly supports our results (40).

This article has several limitations. First, the expression of HCP5 in LSCC tissues and its correlation with clinical features of LSCC were not explored. Second, the effect of HCP5 on LSCC was not performed to verify *in vivo*. In addition, the downstream signaling pathways regulated by ZEB1 in LSCC need to be found in further exploration. Finally, the clinical application of HCP5 still needs to overcome many problems. The entry of all lncRNA-based therapies into the clinic, such as specificity, delivery mode, and immunogenicity (41), has been hindered. The lncRNA may be taken up by other cells than the target cells, resulting in off-target effects and low specificity. The structural instability of lncRNAs leads to low efficiency of intracellular delivery of lncRNA; in addition, exogenous lncRNAs are prone to lead to tolerance problems (immunogenicity problems).

## Conclusion

**Figure 7:**
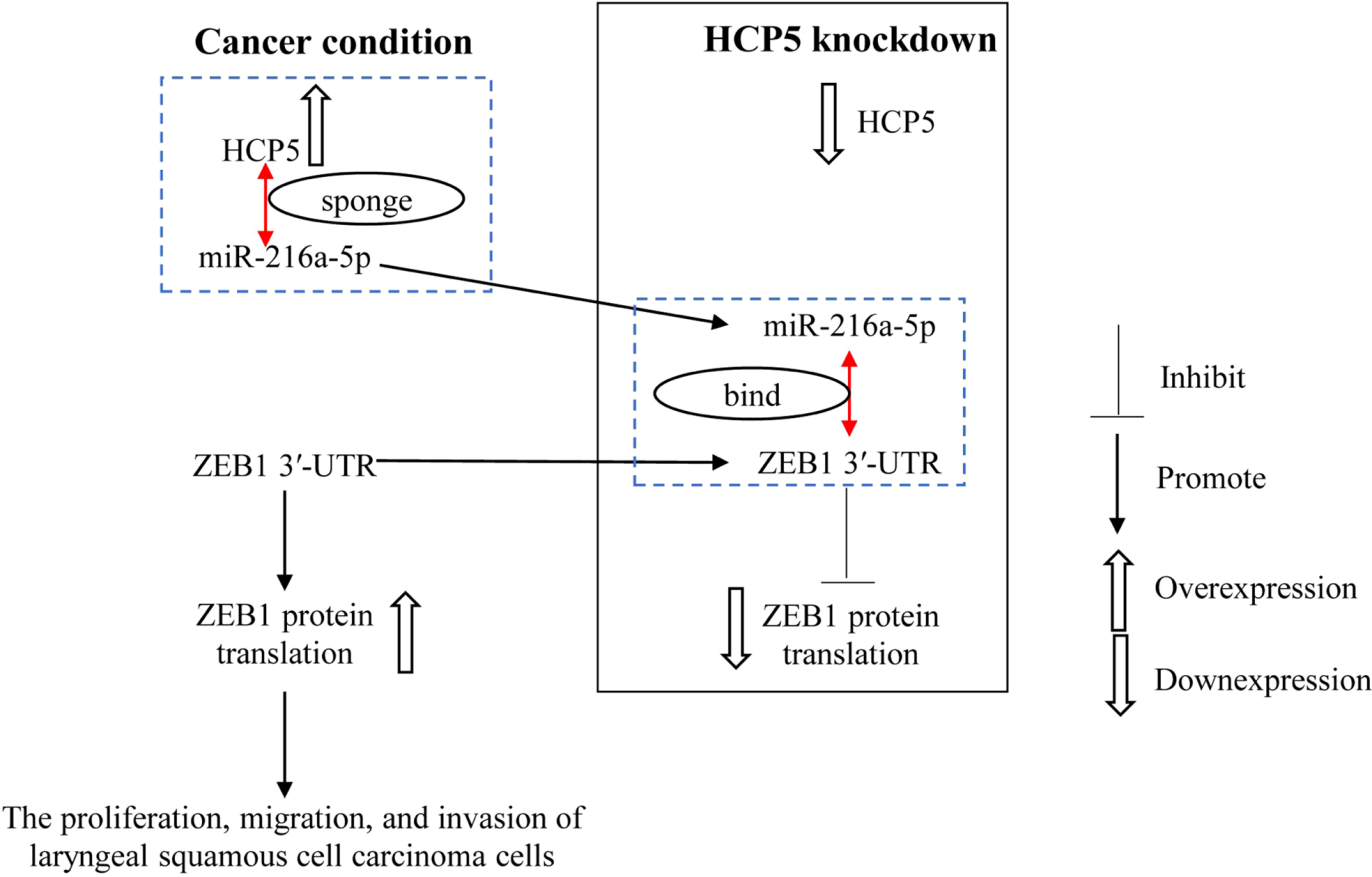
miR-216a-5p binds with ZEB1 3′-UTR to inhibit ZEB1 expression. HCP5 enhances the malignant biological function of LSCC cells by sponging miR-216a-5p to promote ZEB1 expression.

HCP5 promotes the proliferation, migration, and invasion of LSCC by regulating miR-216a-5p/ZEB1, and HCP5 knockdown exerts the opposite effect (**Figure 7**), suggesting that HCP5 may be a prospective therapeutic target for LSCC. The therapeutic efficacy of HCP5 on LSCC needs to be validated by more *in vivo* and clinical investigations.

## Data availability statement

The original contributions presented in the study are included in the article/**Supplementary Material**. Further inquiries can be directed to the corresponding author.

## Author contributions

SZ: Conceptualization, Data Curation, Visualization, and Writing - Original Draft; HH and QZ: Conceptualization, Formal analysis, and Writing - Review and Editing YL: Project administration and Data Curation; LW : Investigation and Data Curation and Writing - Review and Editing. All authors contributed to the article and approved the submitted version.

## Funding

This study was supported by Science Foundation for Youths of Shanxi Province of China (No. 201601D021140) Natural Science Foundation of Basic research program of Shanxi Province, China (No. 20210302123250) and Startup Foundation for Doctors of Shanxi Medical University (No. 03201628).

## Conflict of interest

The authors declare that the research was conducted in the absence of any commercial or financial relationships that could be construed as a potential conflict of interest.

## Publisher’s note

All claims expressed in this article are solely those of the authors and do not necessarily represent those of their affiliated organizations, or those of the publisher, the editors and the reviewers. Any product that may be evaluated in this article, or claim that may be made by its manufacturer, is not guaranteed or endorsed by the publisher.

## Abbreviations

LSCC, laryngeal squamous cell carcinoma; lncRNA, long non-coding RNA; HCP5, lncRNA human leukocyte antigen complex P5; miRNA, microRNA; siRNA, small interference RNAs; ANOVA, One-way analysis of variance.
