# Supplementary figures and images for: Downregulation of long noncoding RNA HCP5/miR-216a-5p/ZEB1 axis inhibits the malignant biological function of laryngeal squamous cell carcinoma cells
Source: Front Immunol. 2022 Sep 30;13:1022677. doi: 10.3389/fimmu.2022.1022677 (PMC9561619; doi:10.3389/fimmu.2022.1022677)

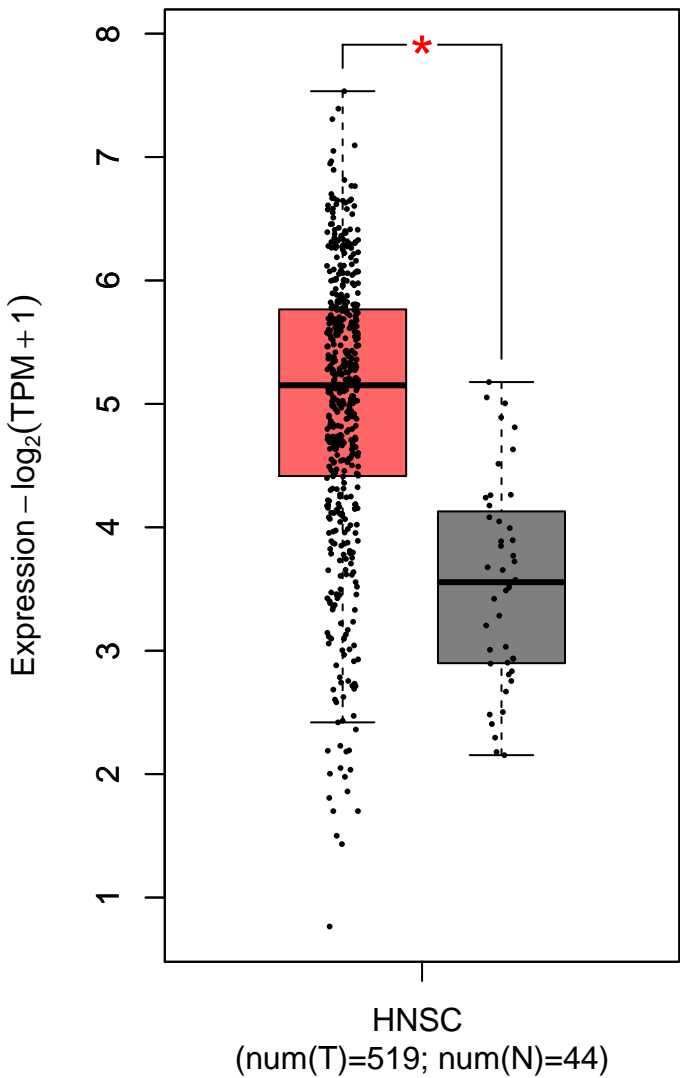

Supplement: Supplementary file 1 [file DataSheet_1.pdf]

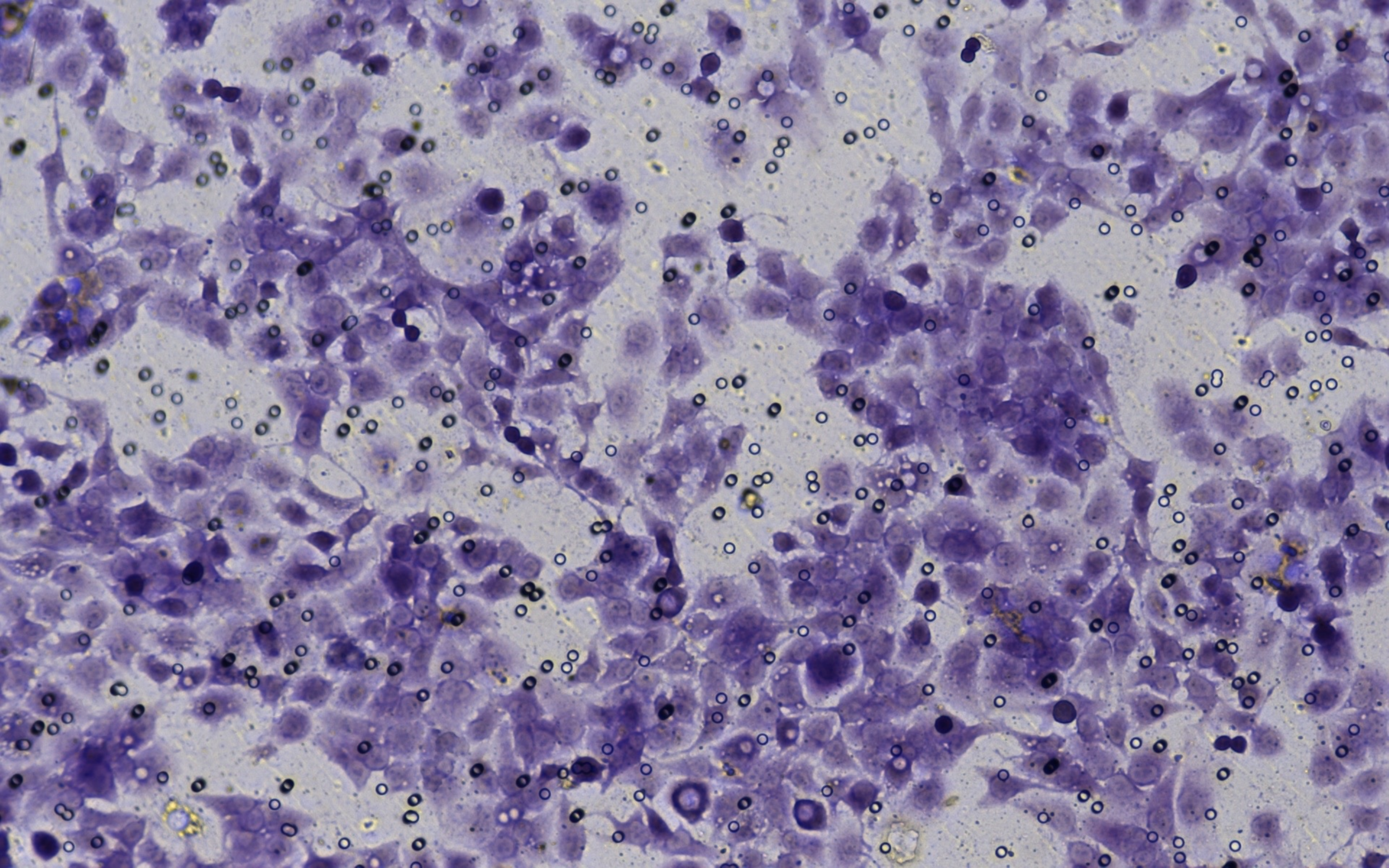

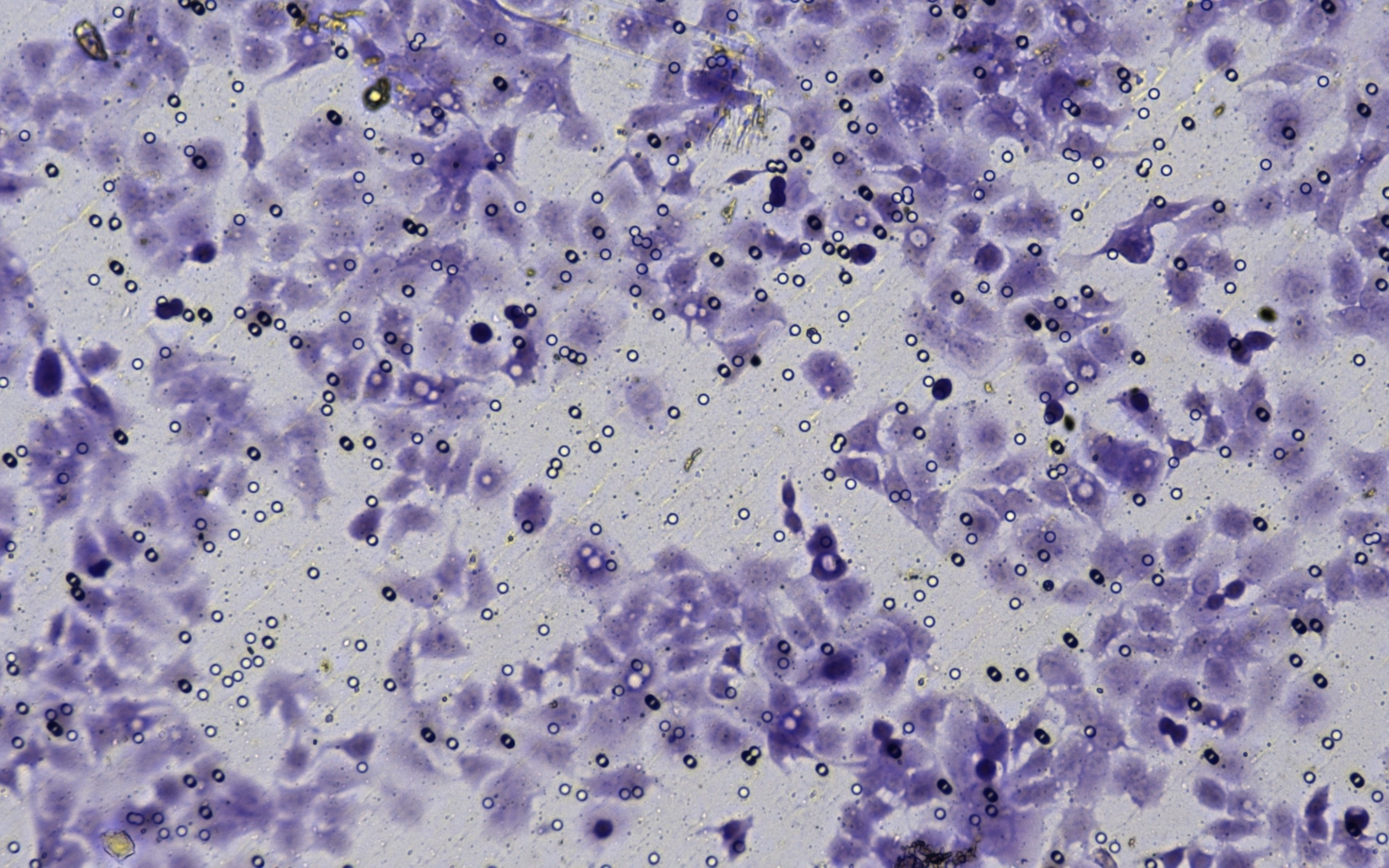

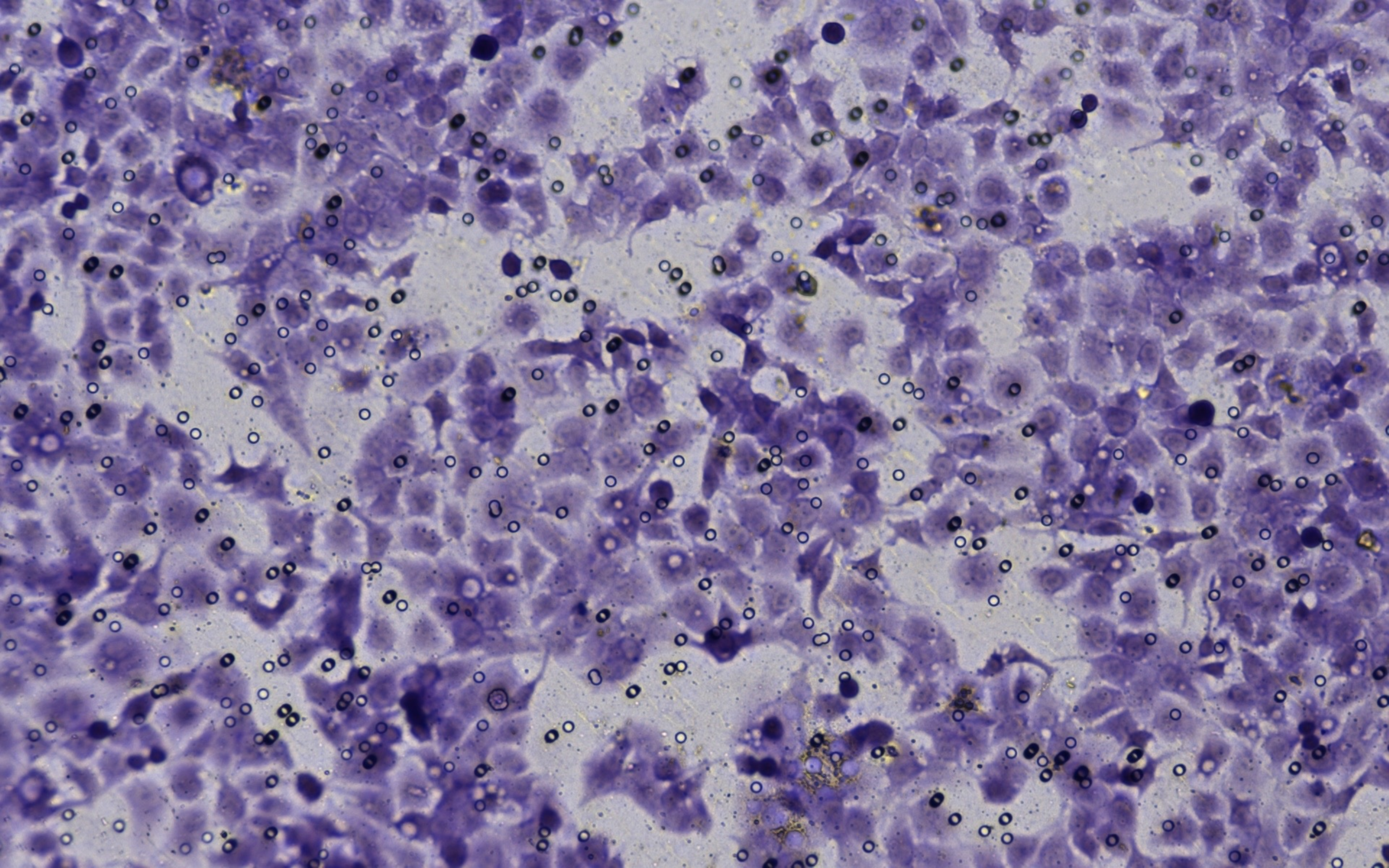

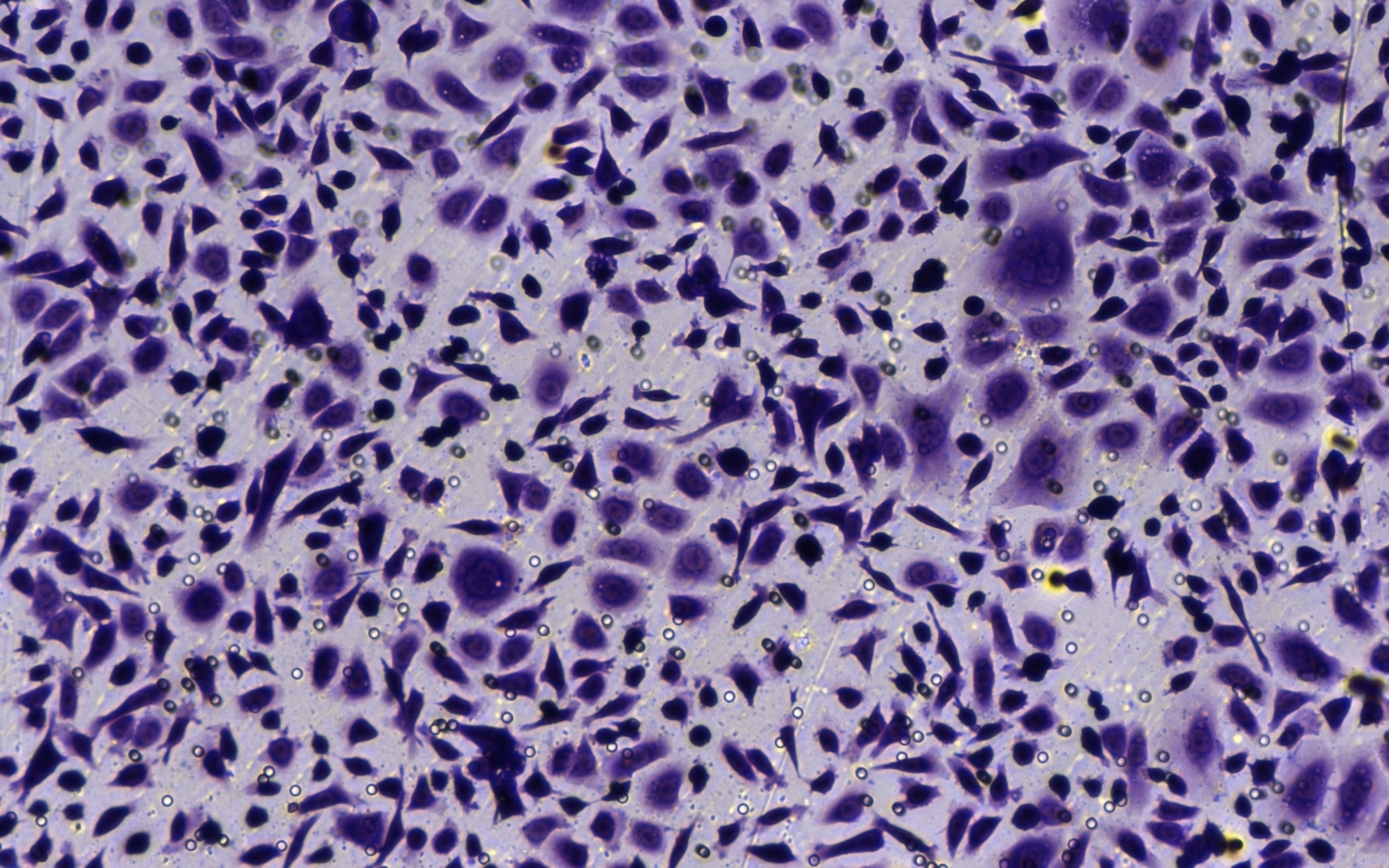

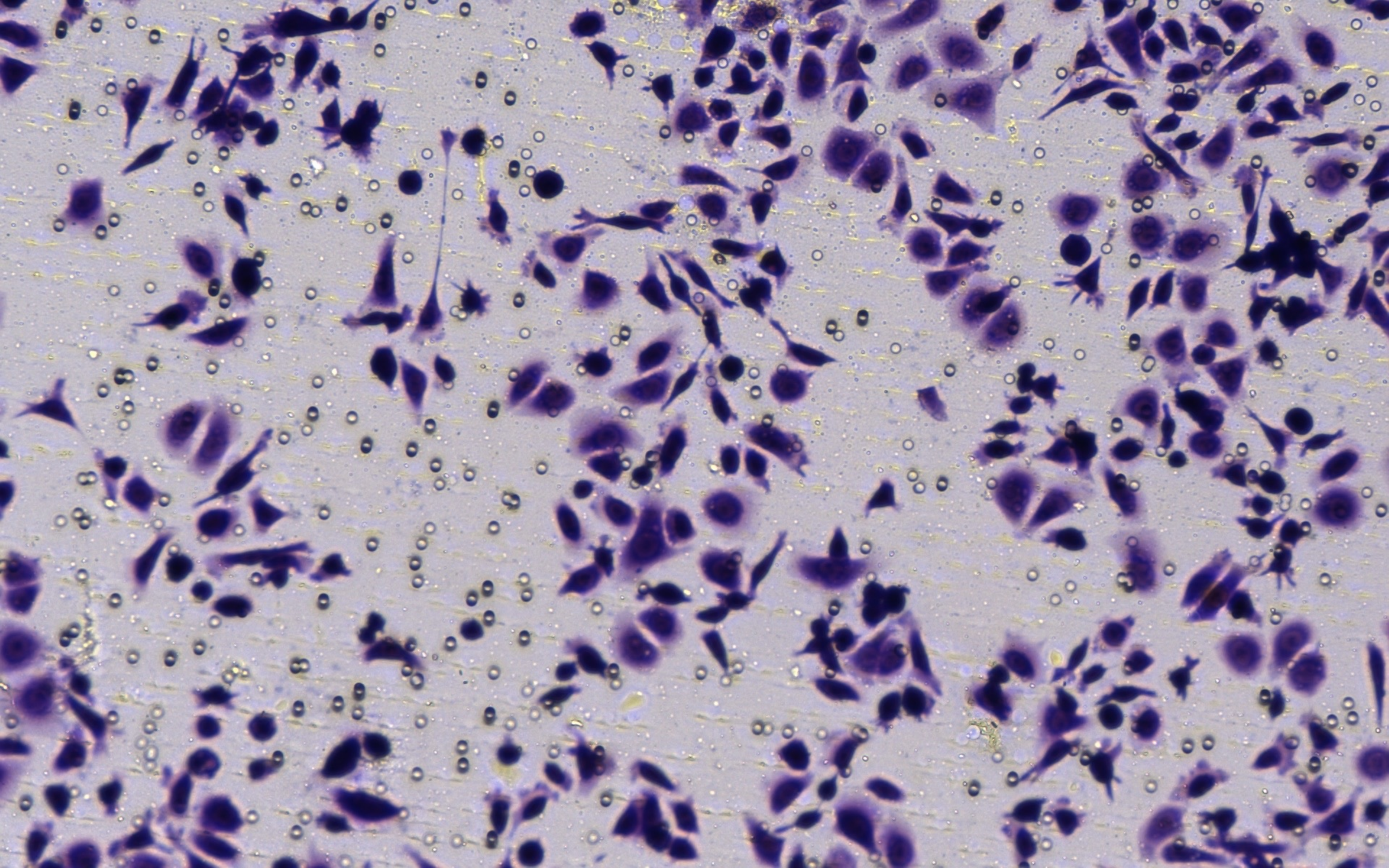

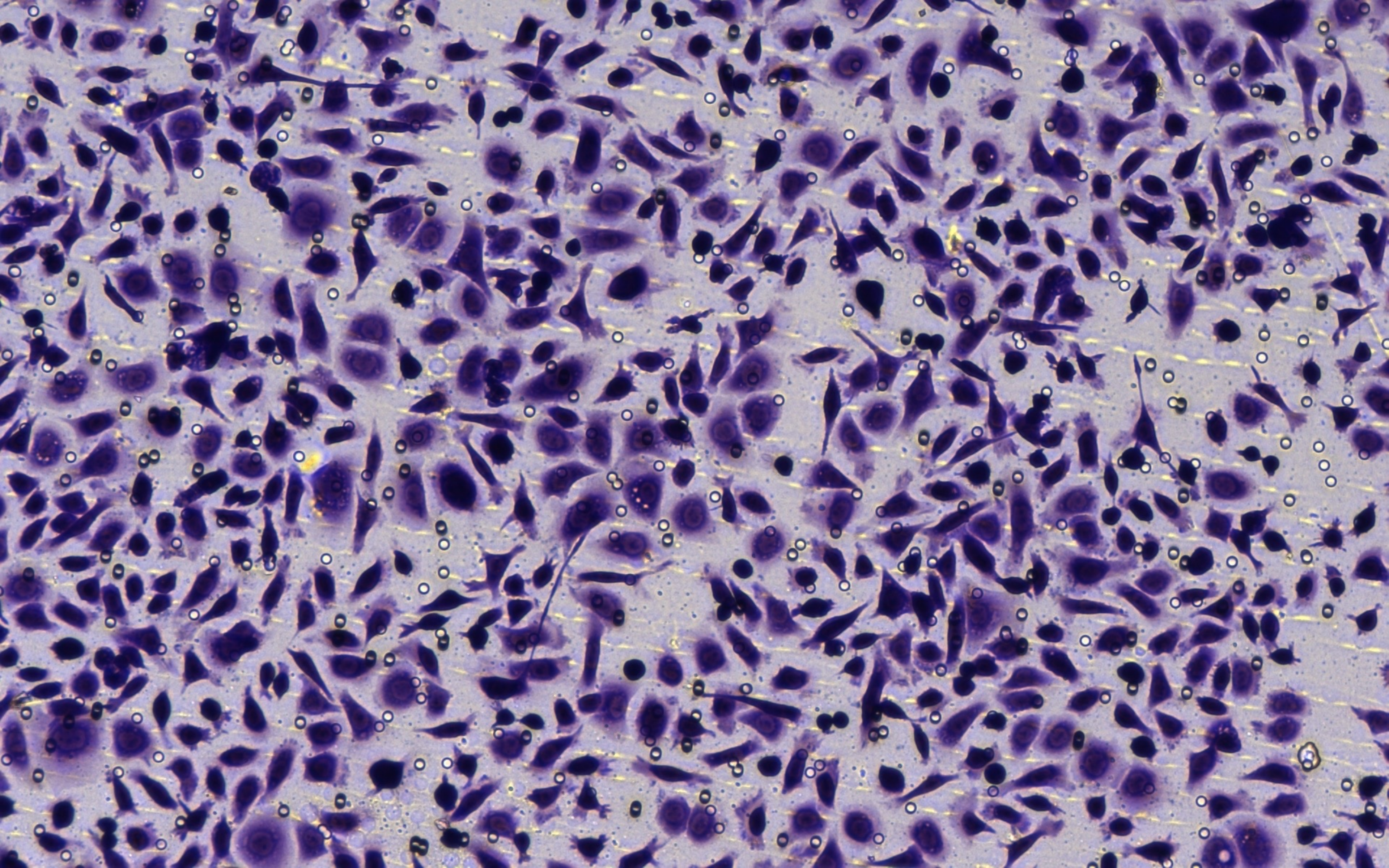

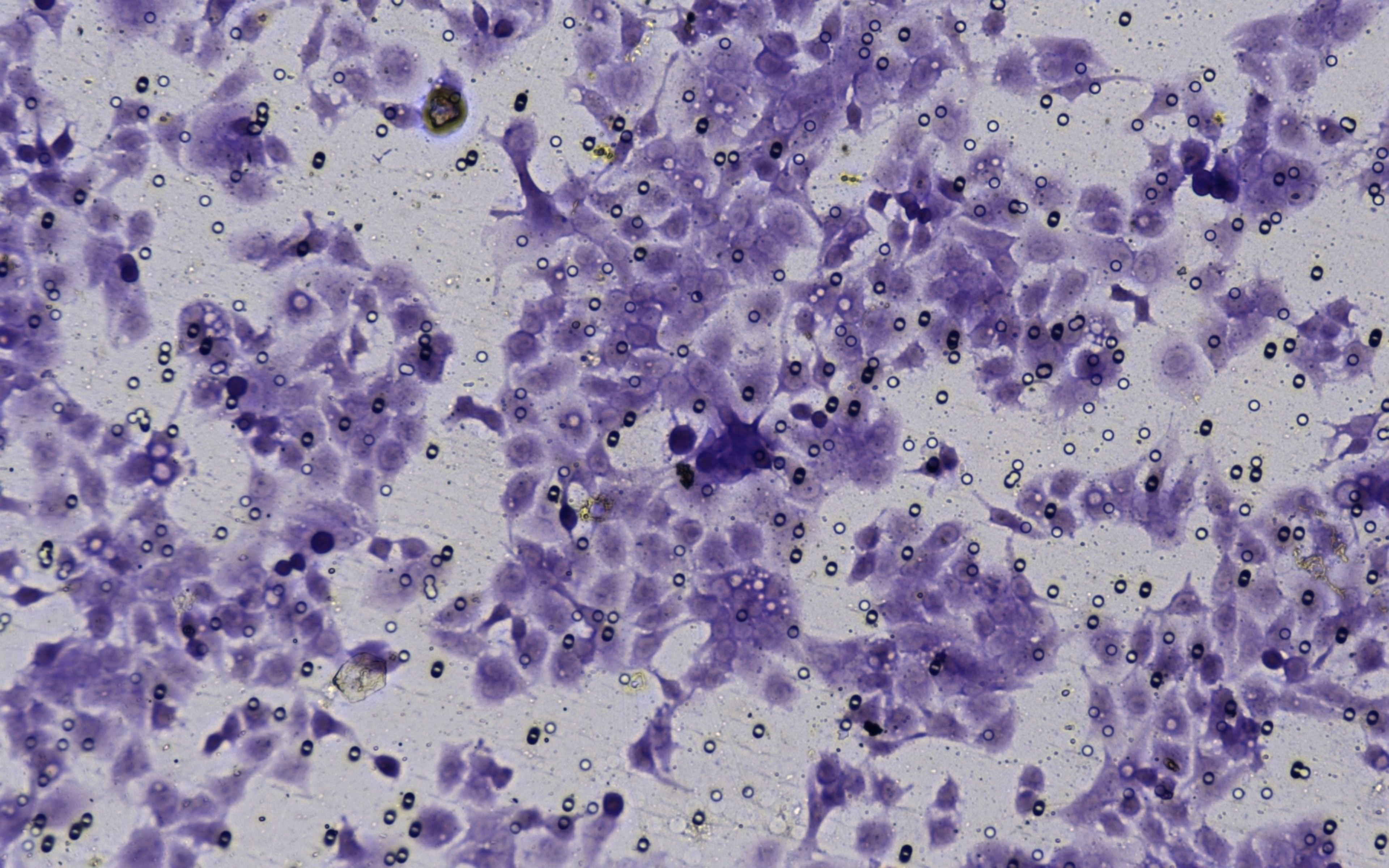

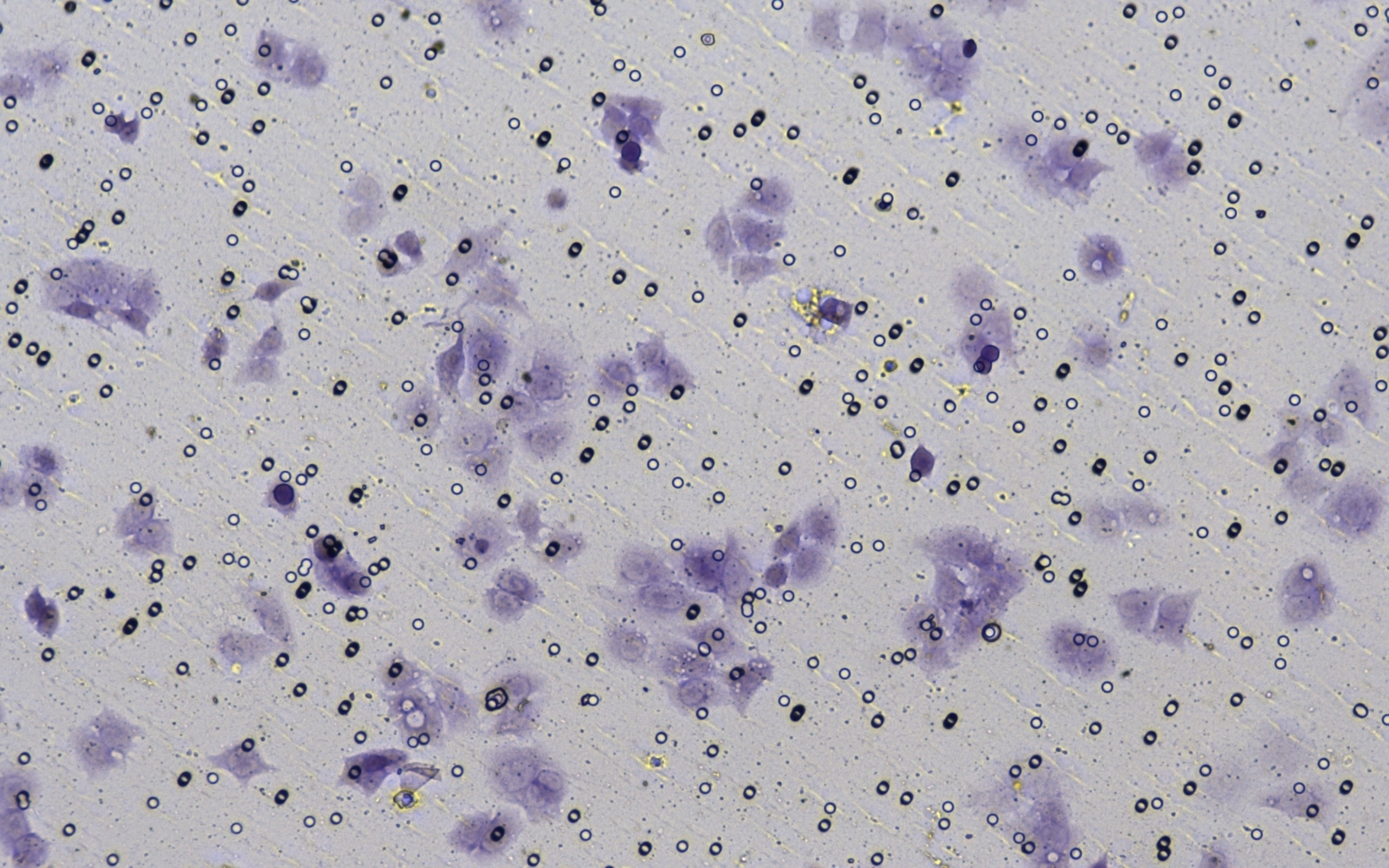

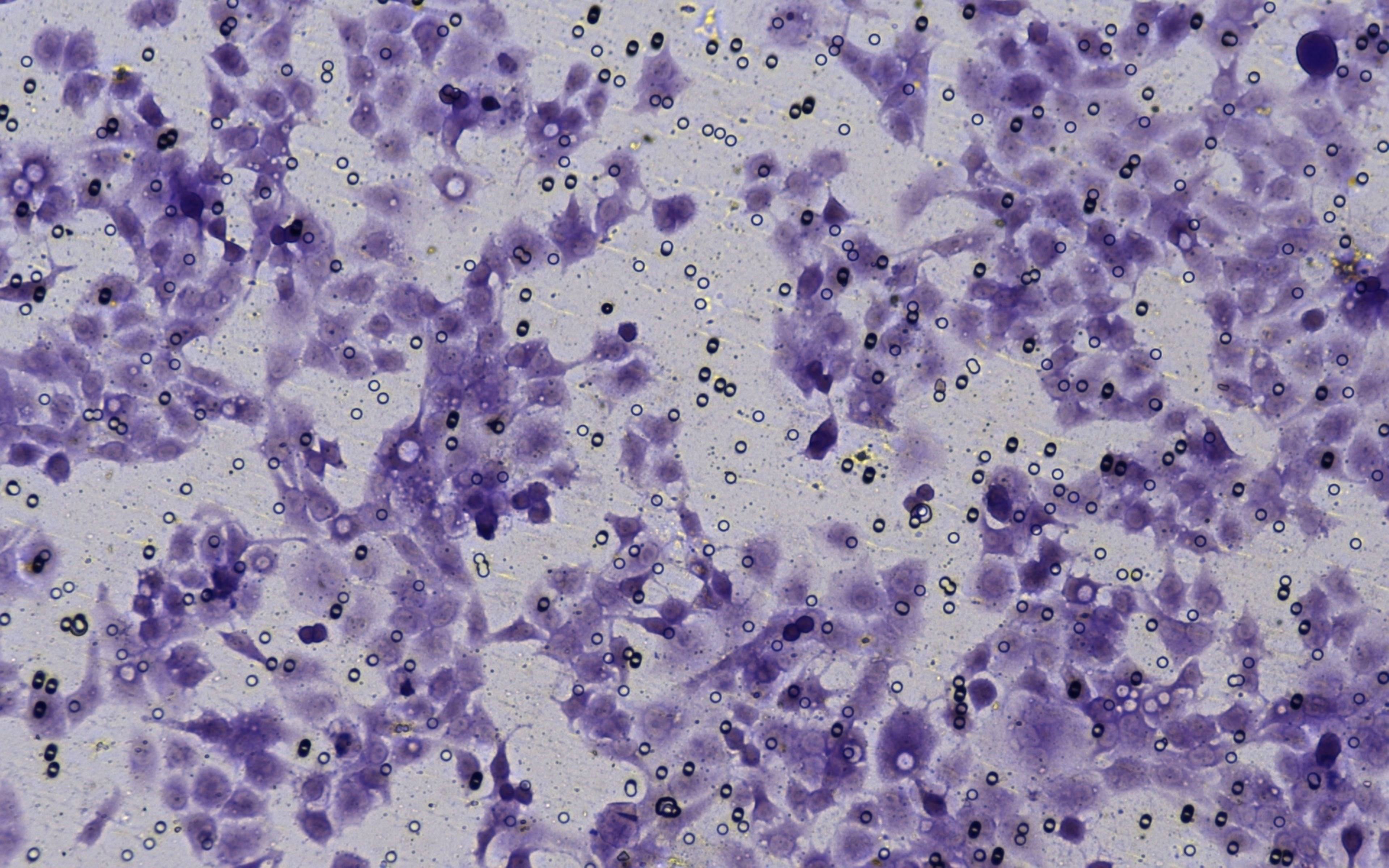

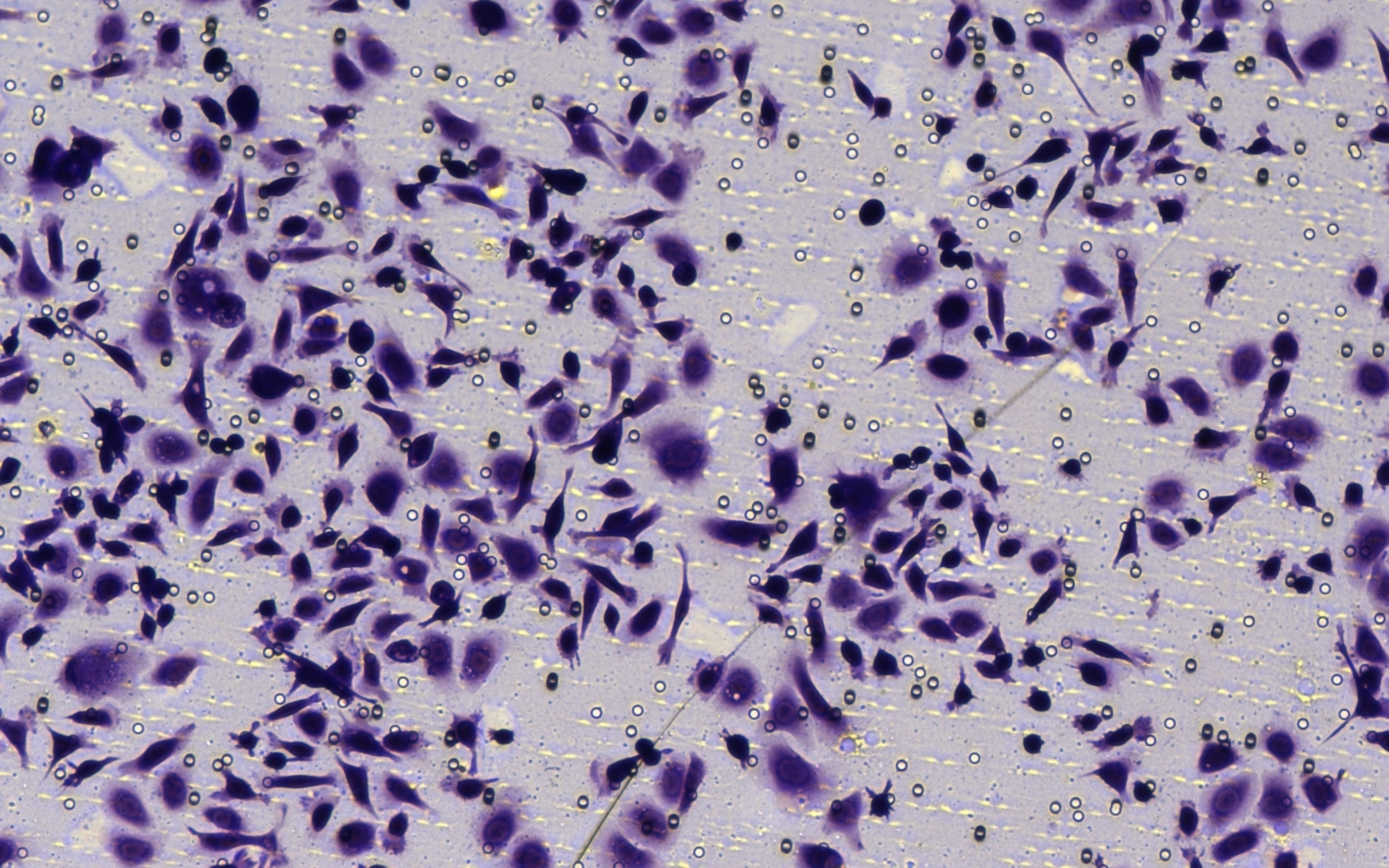

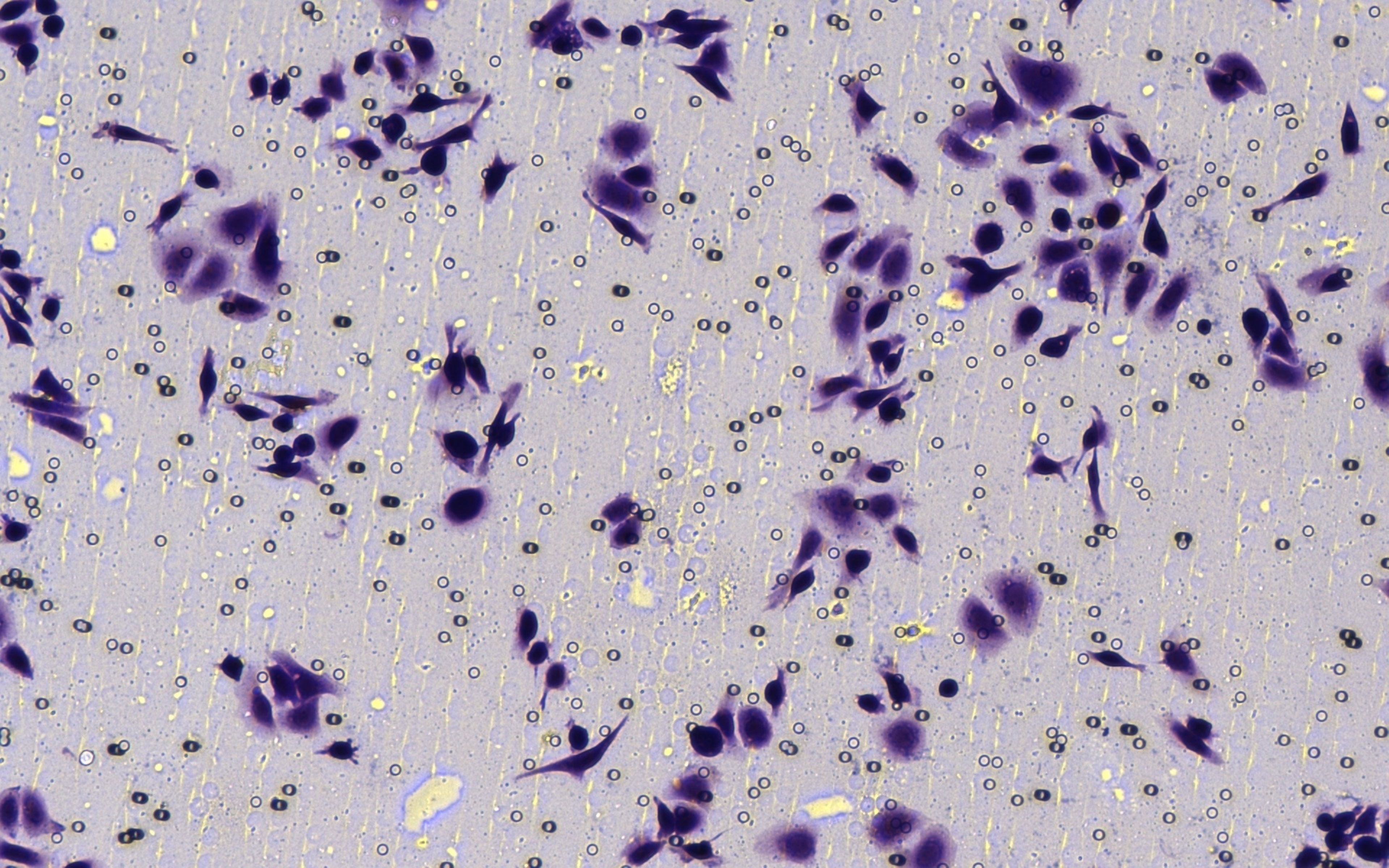

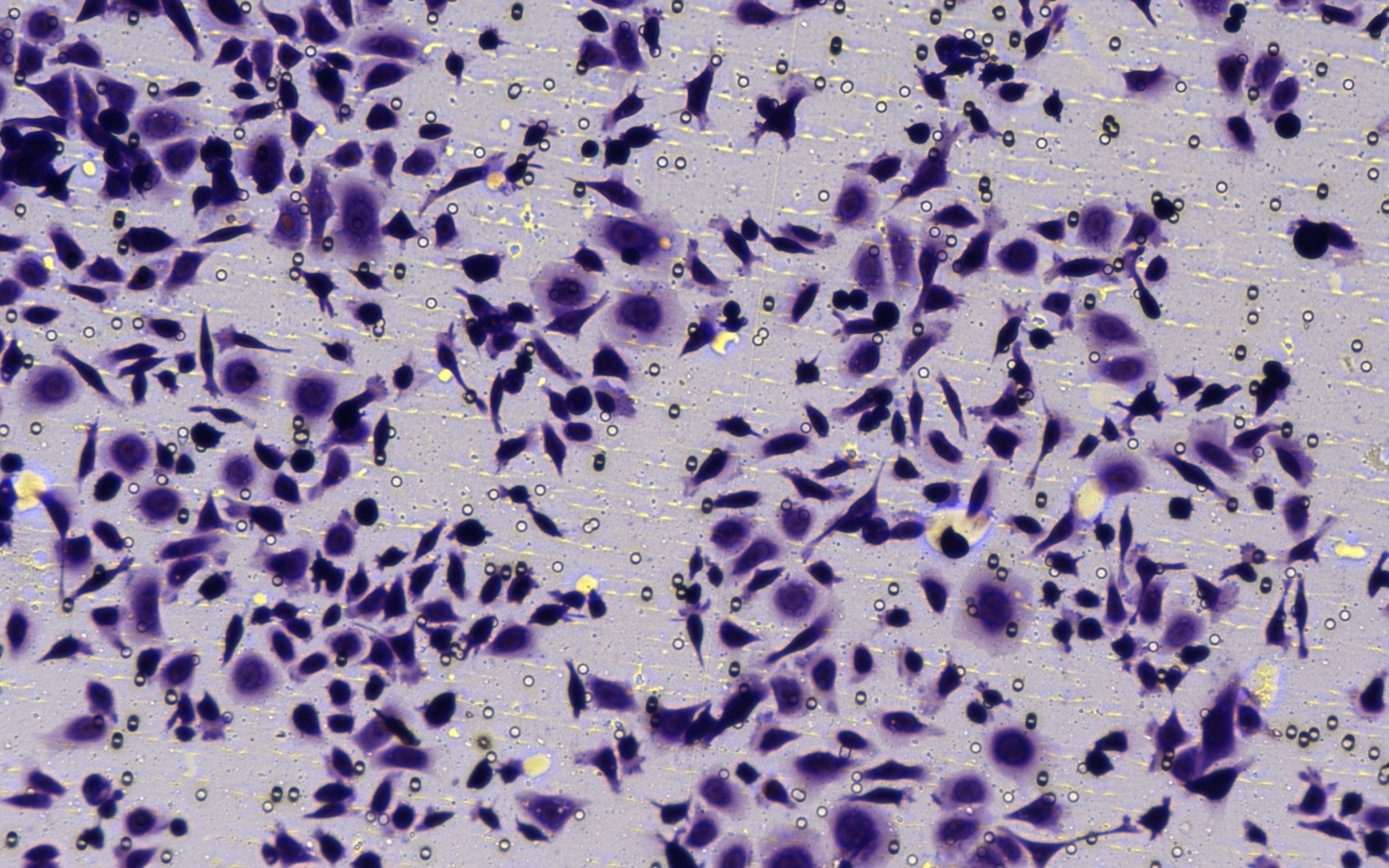

Supplement: Supplementary file 2 [file DataSheet_2.pdf]

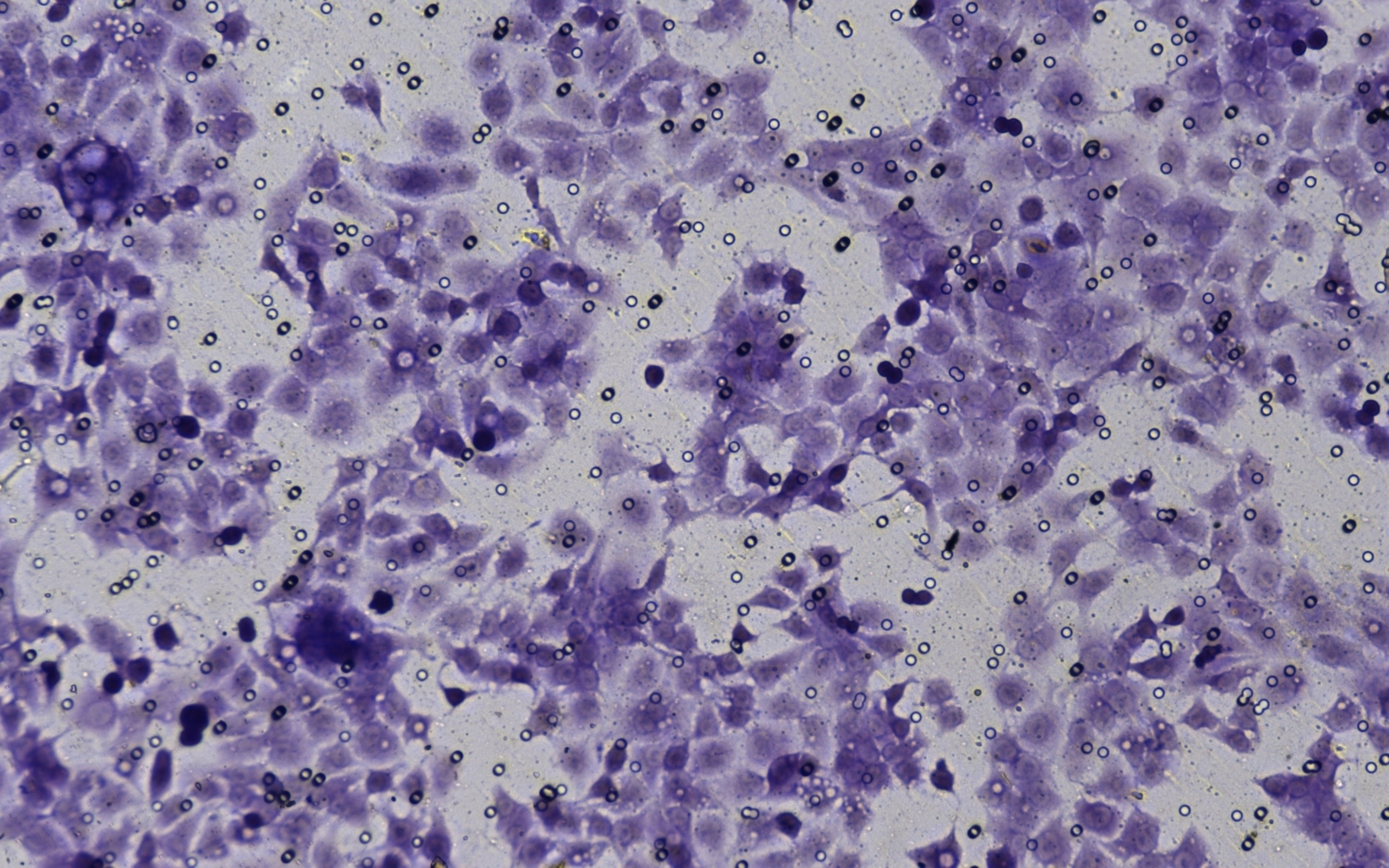

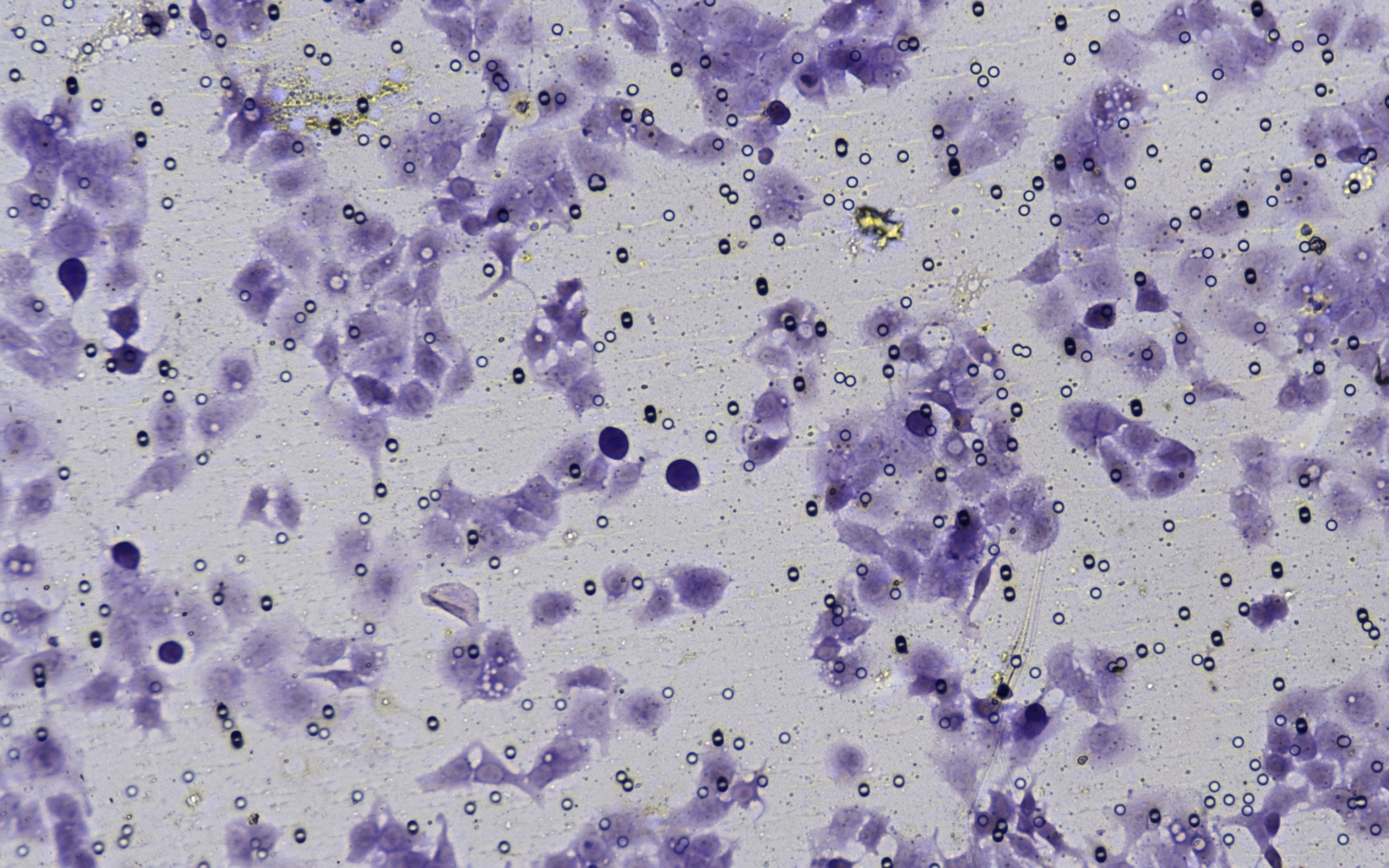

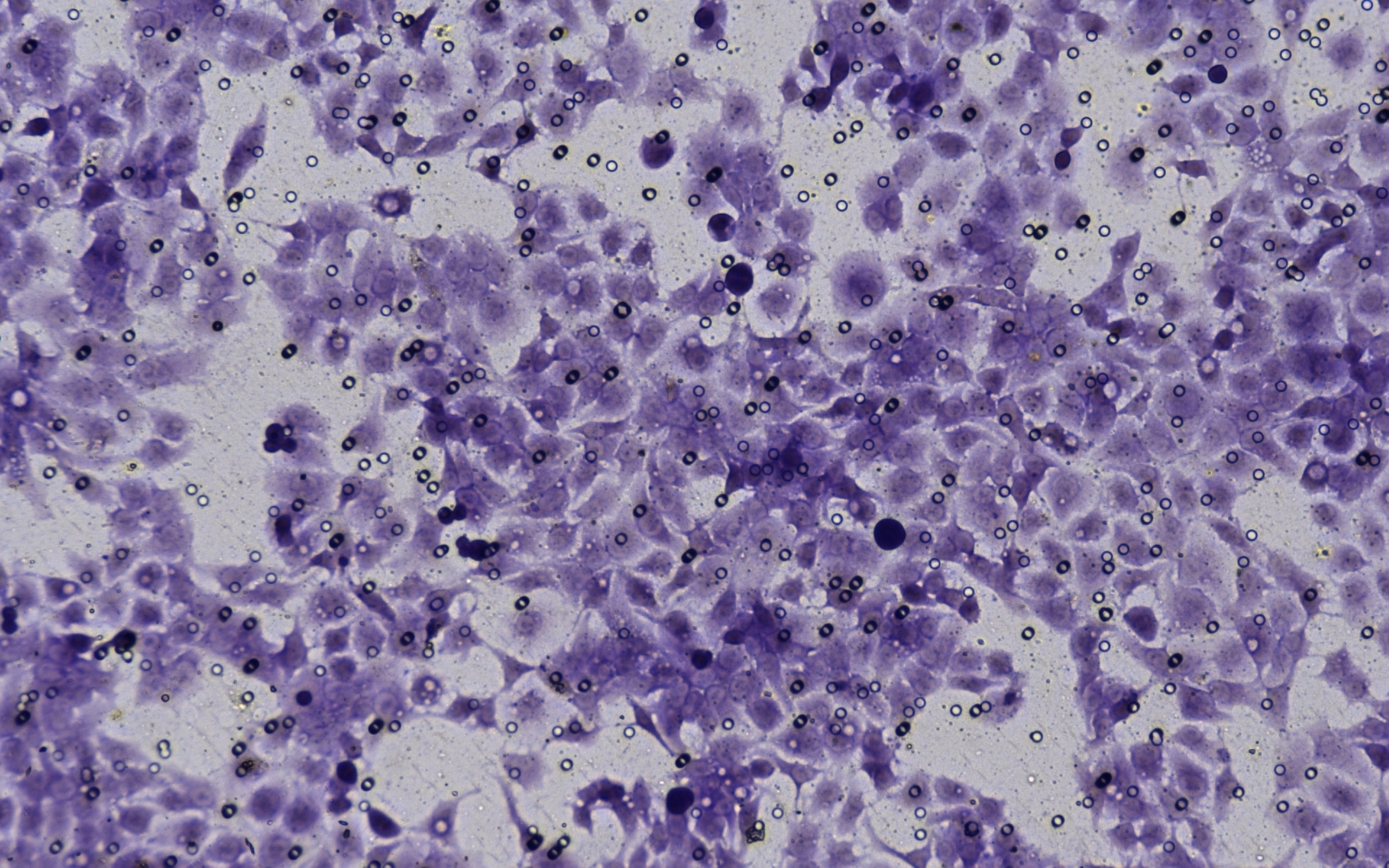

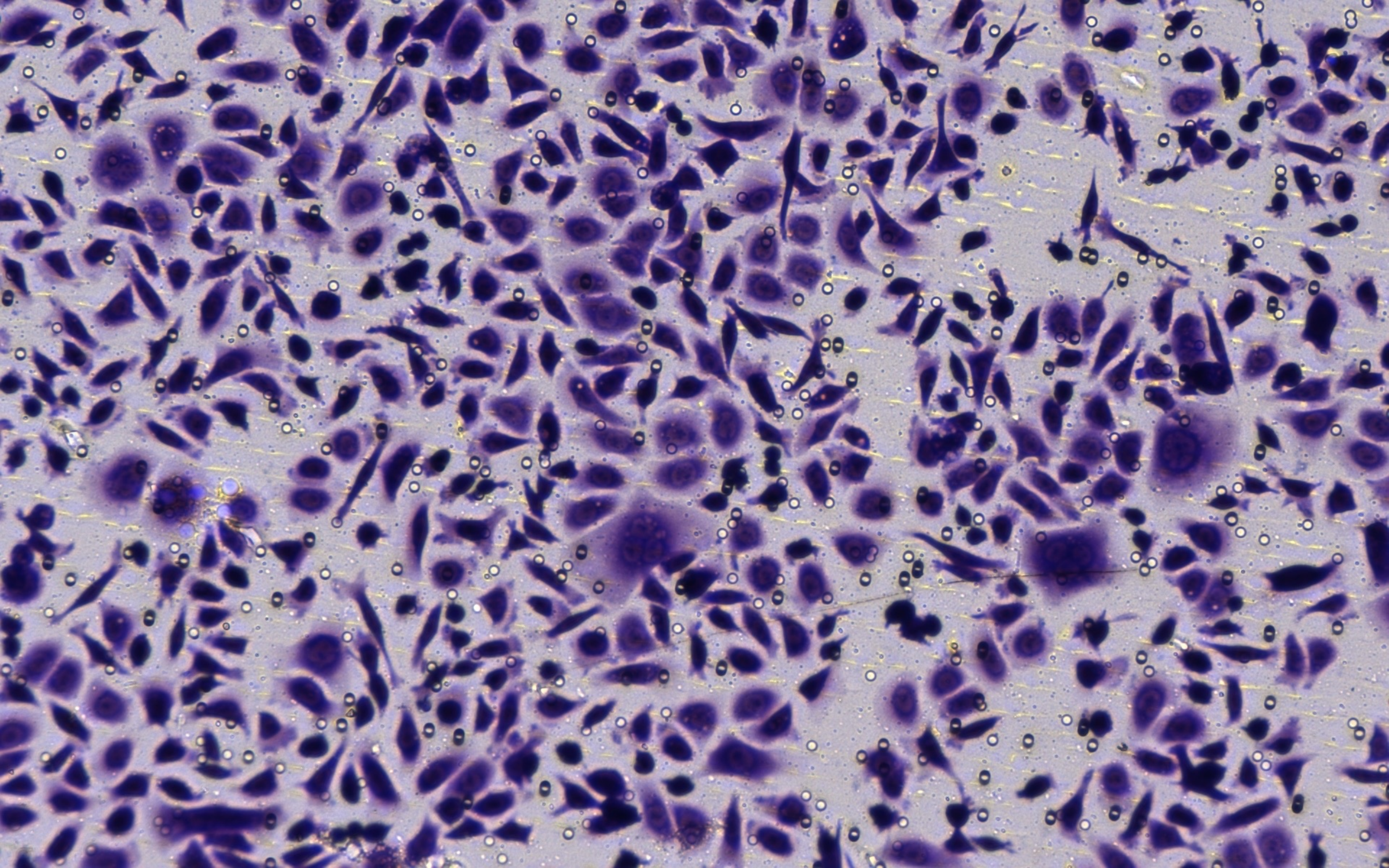

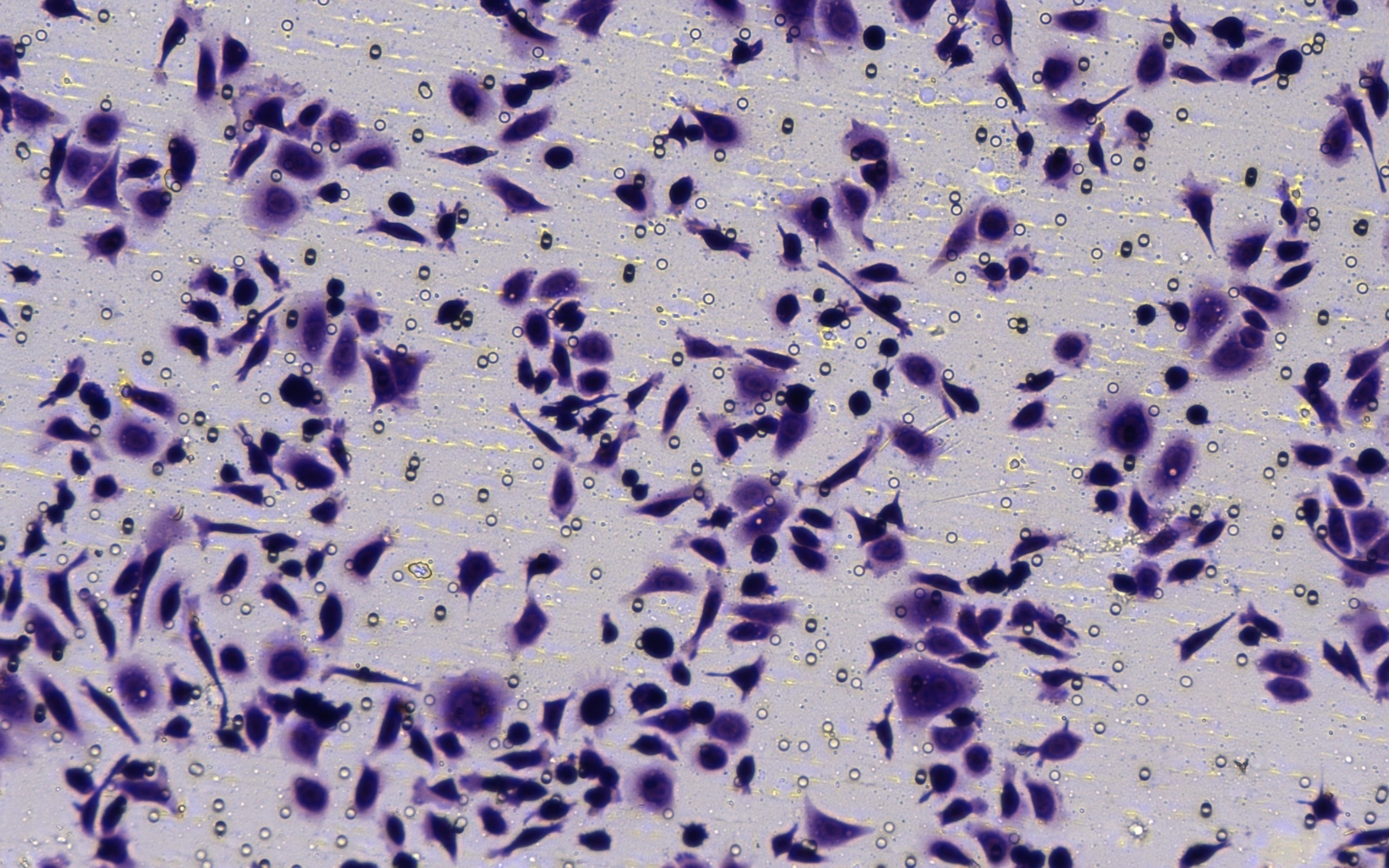

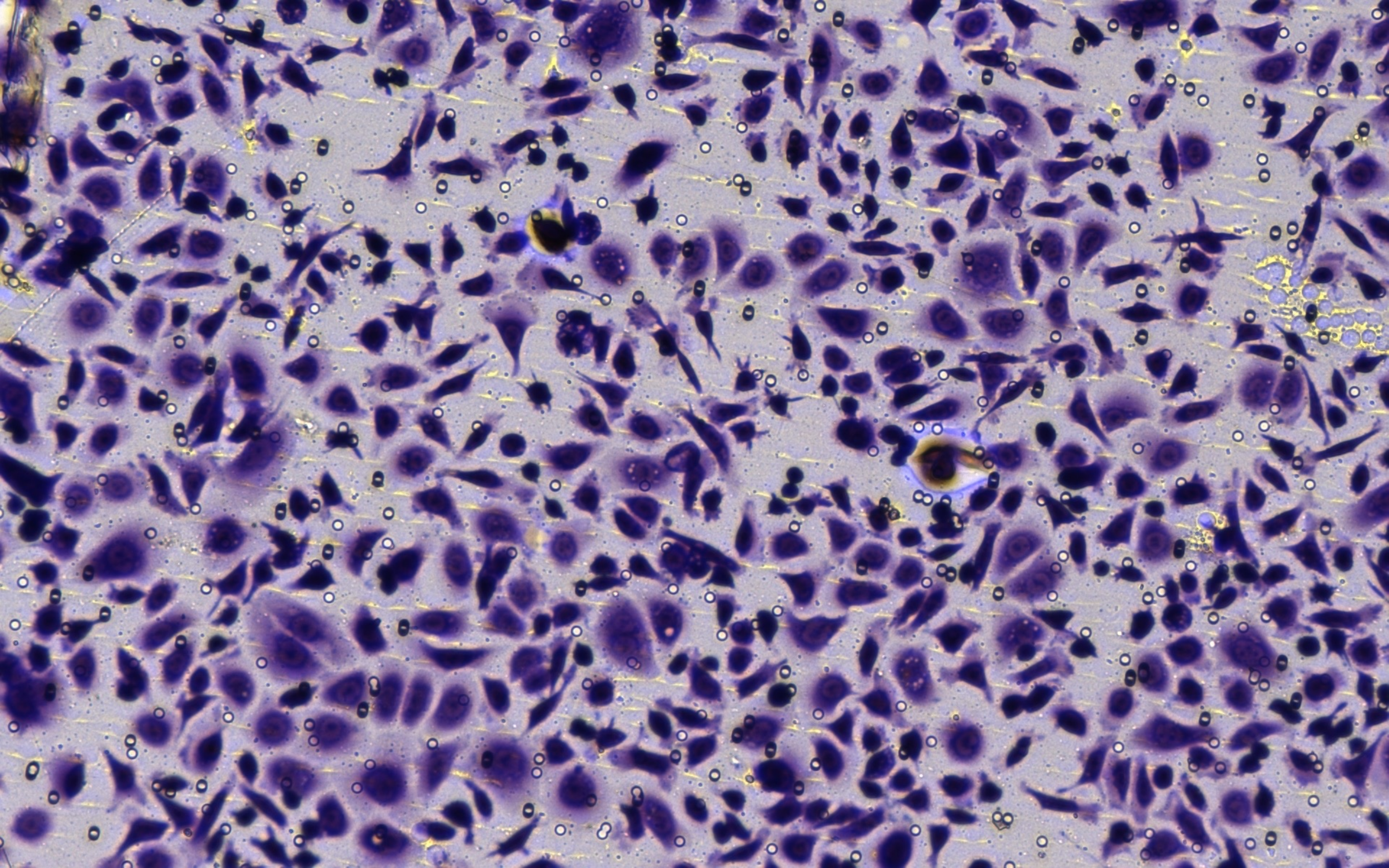

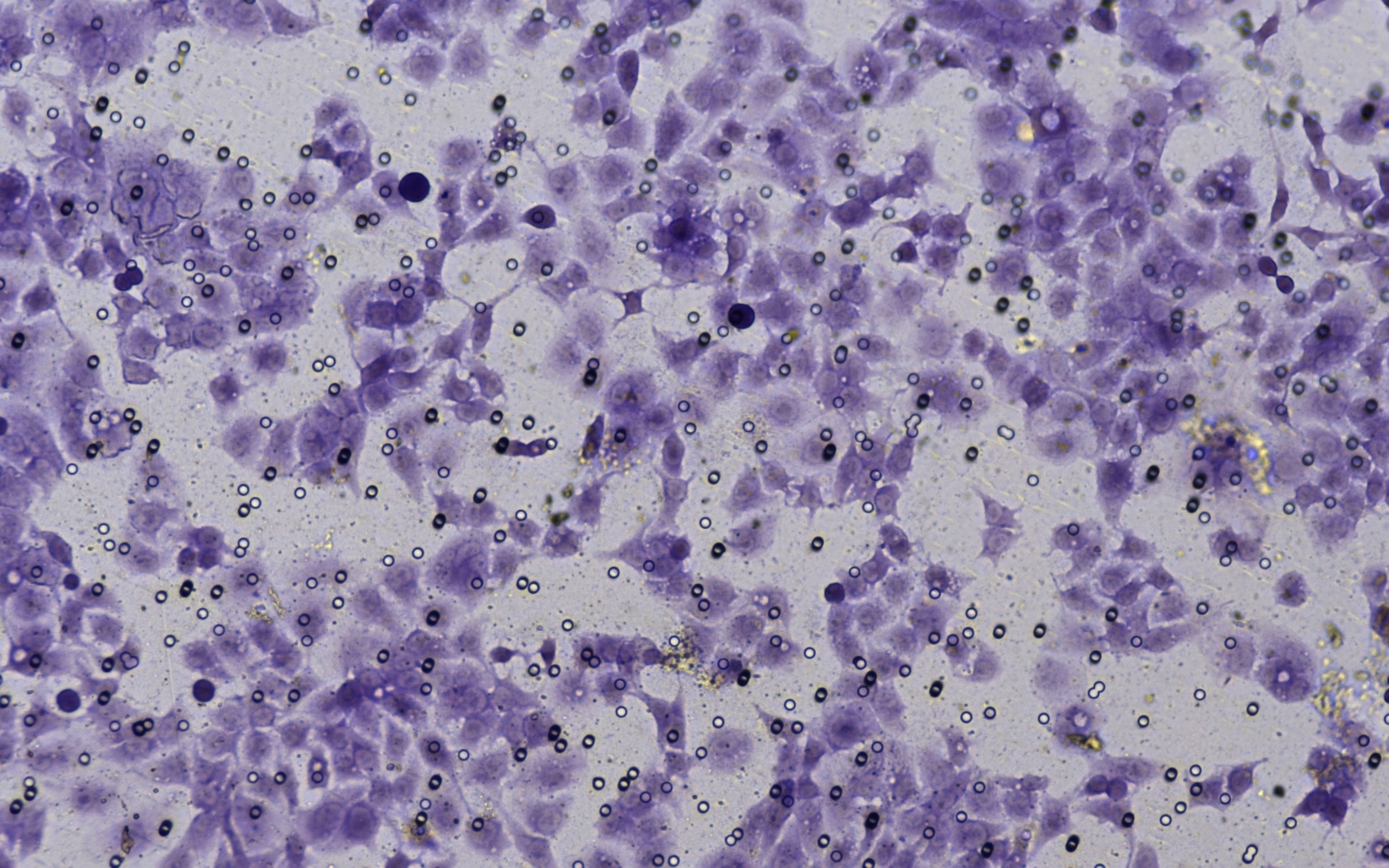

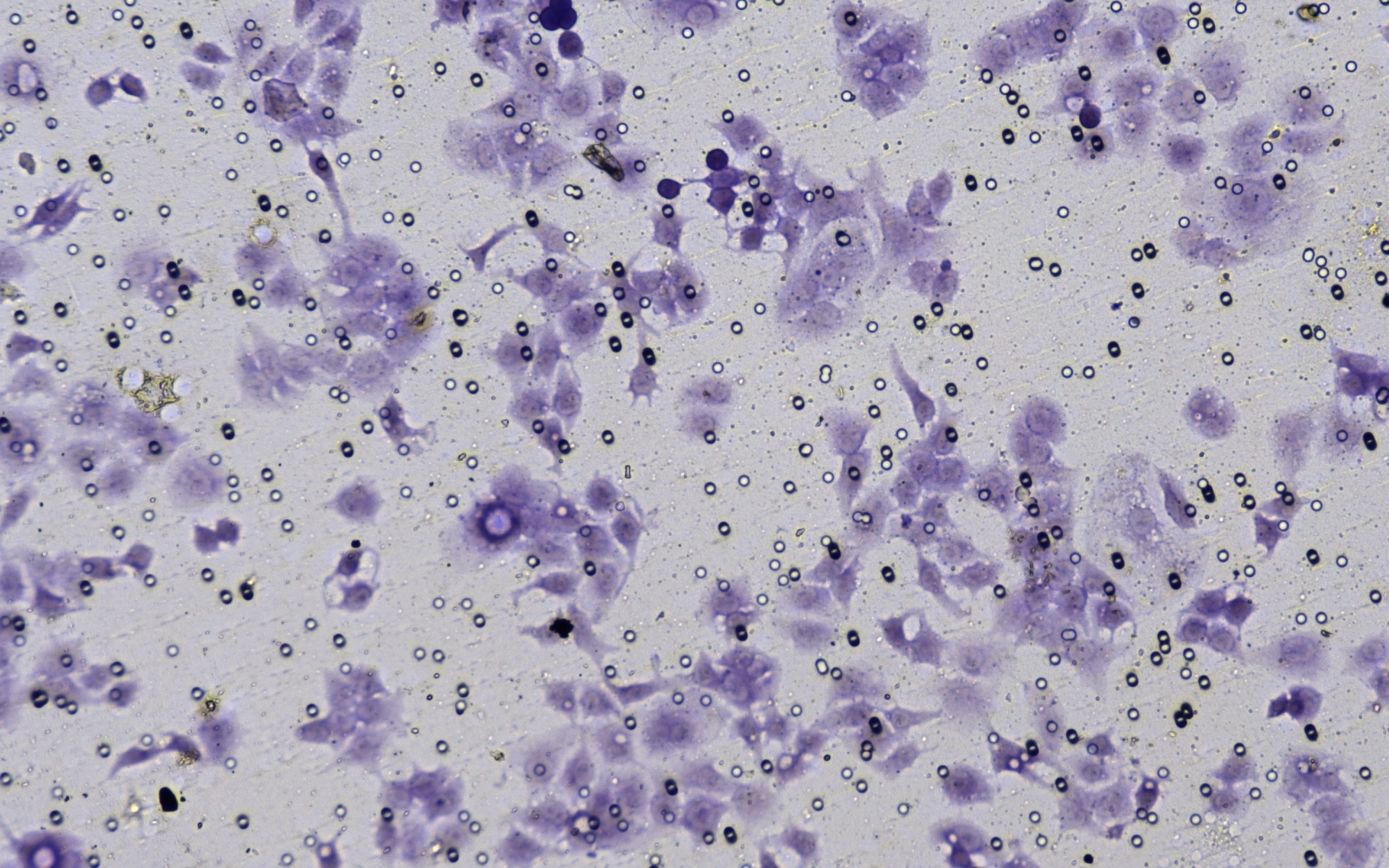

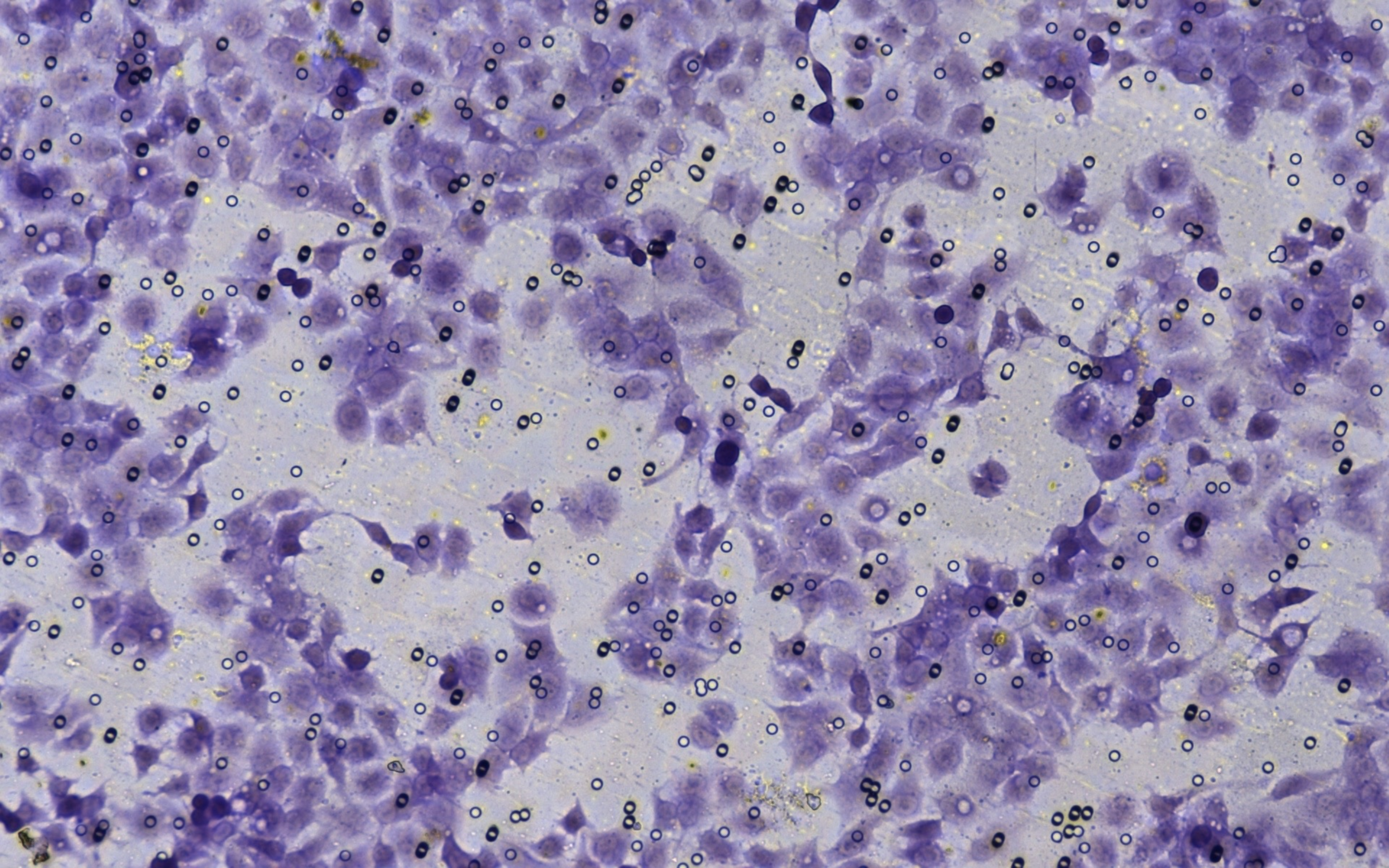

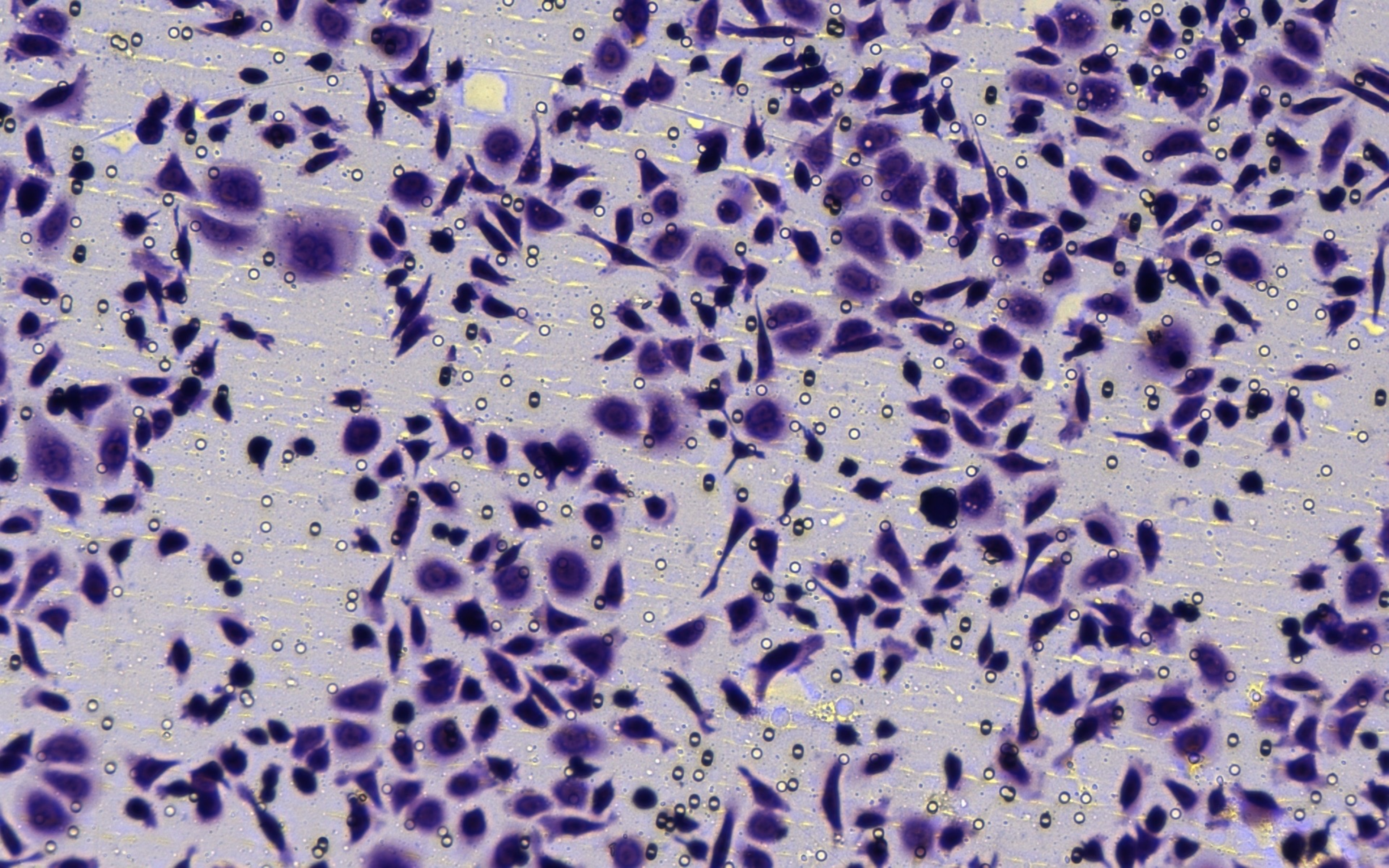

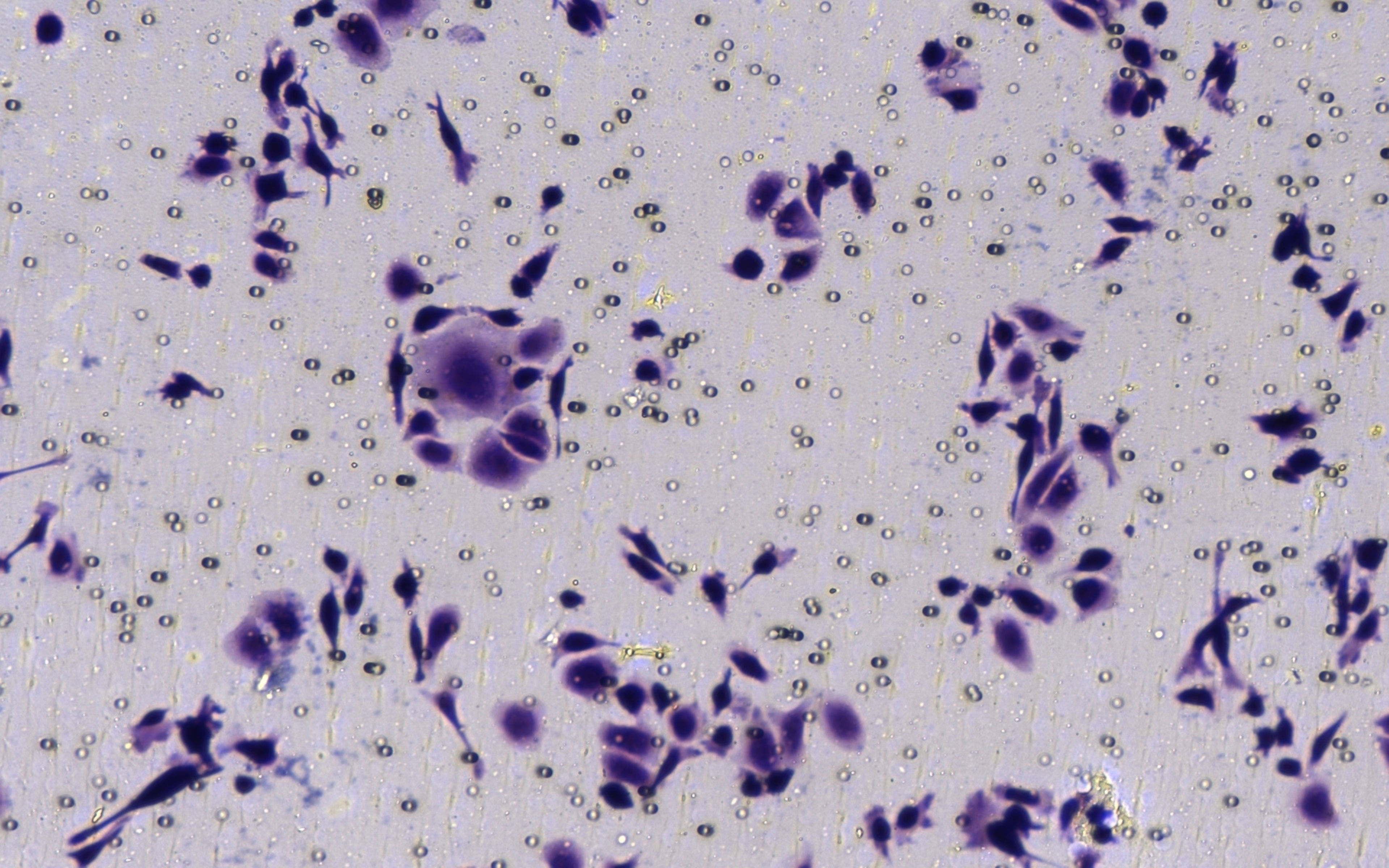

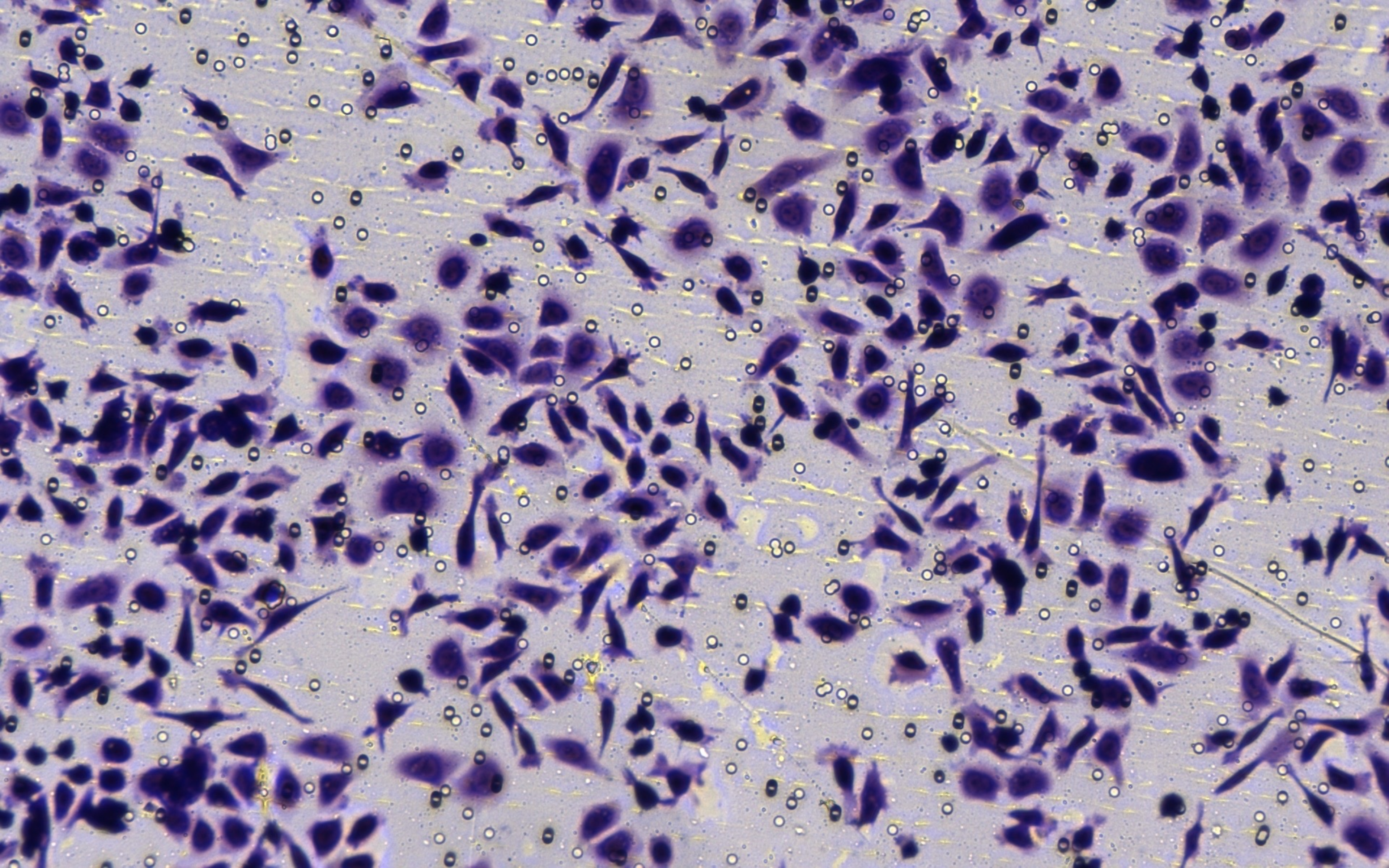

Supplement: Supplementary file 3 [file DataSheet_3.pdf]

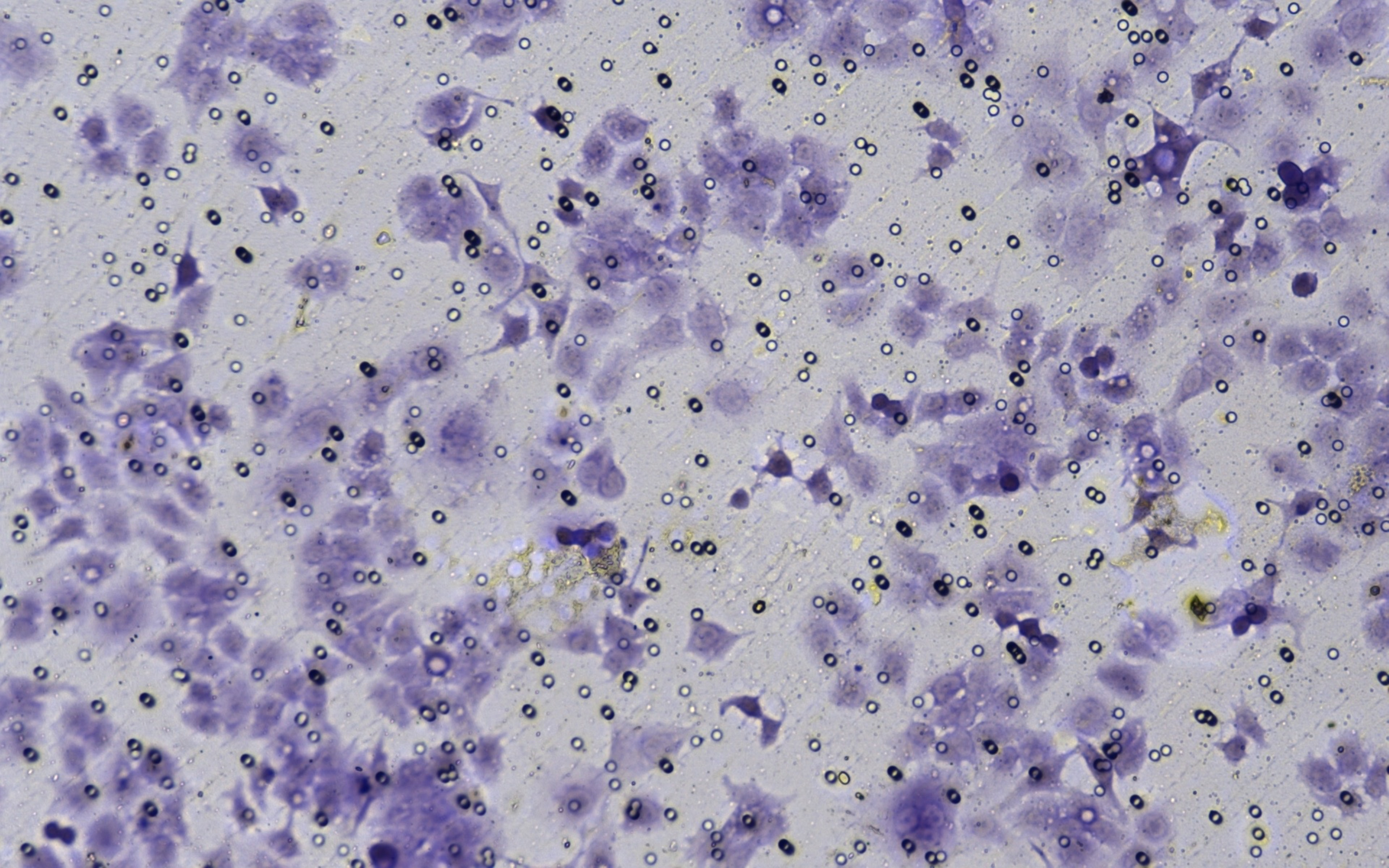

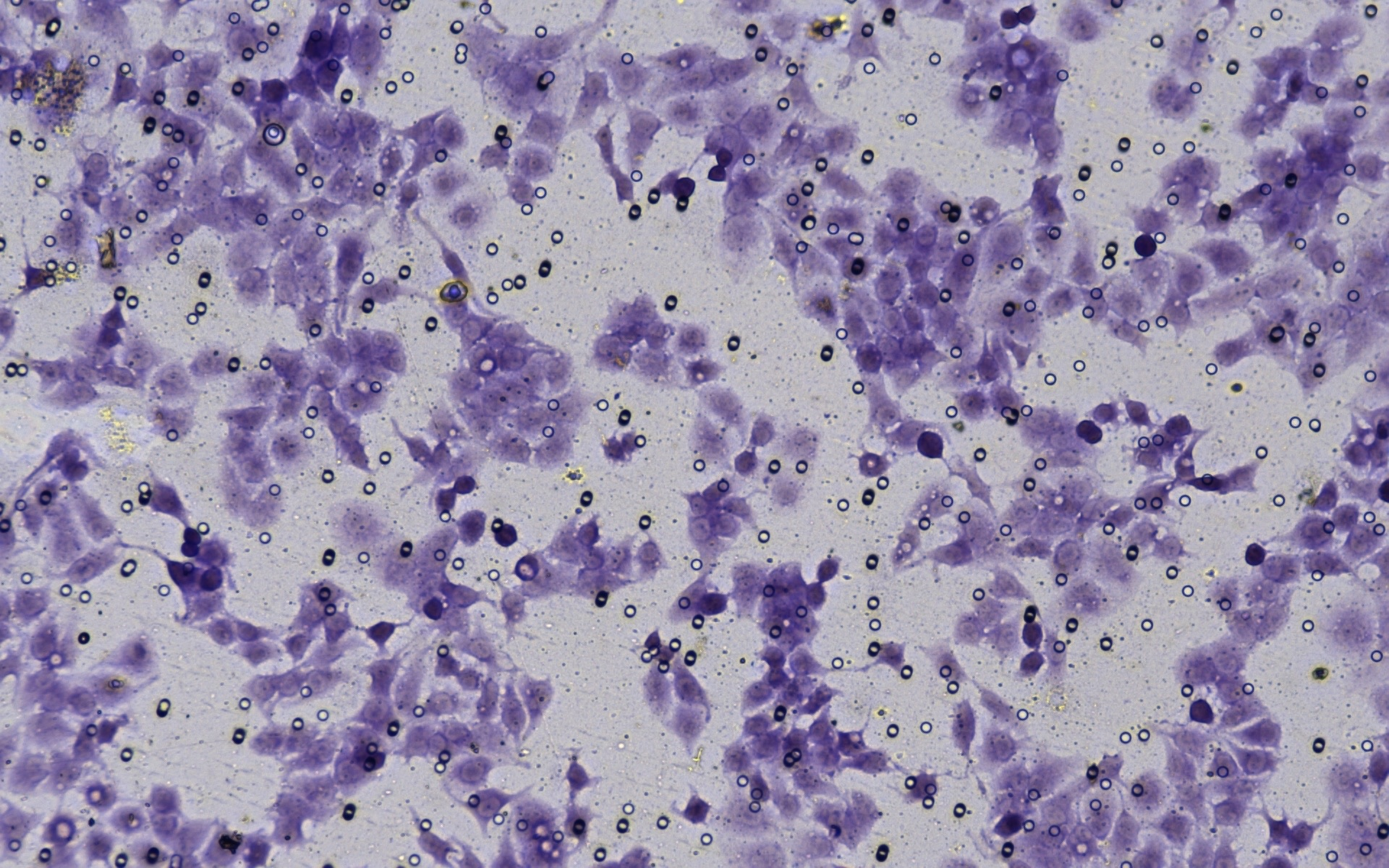

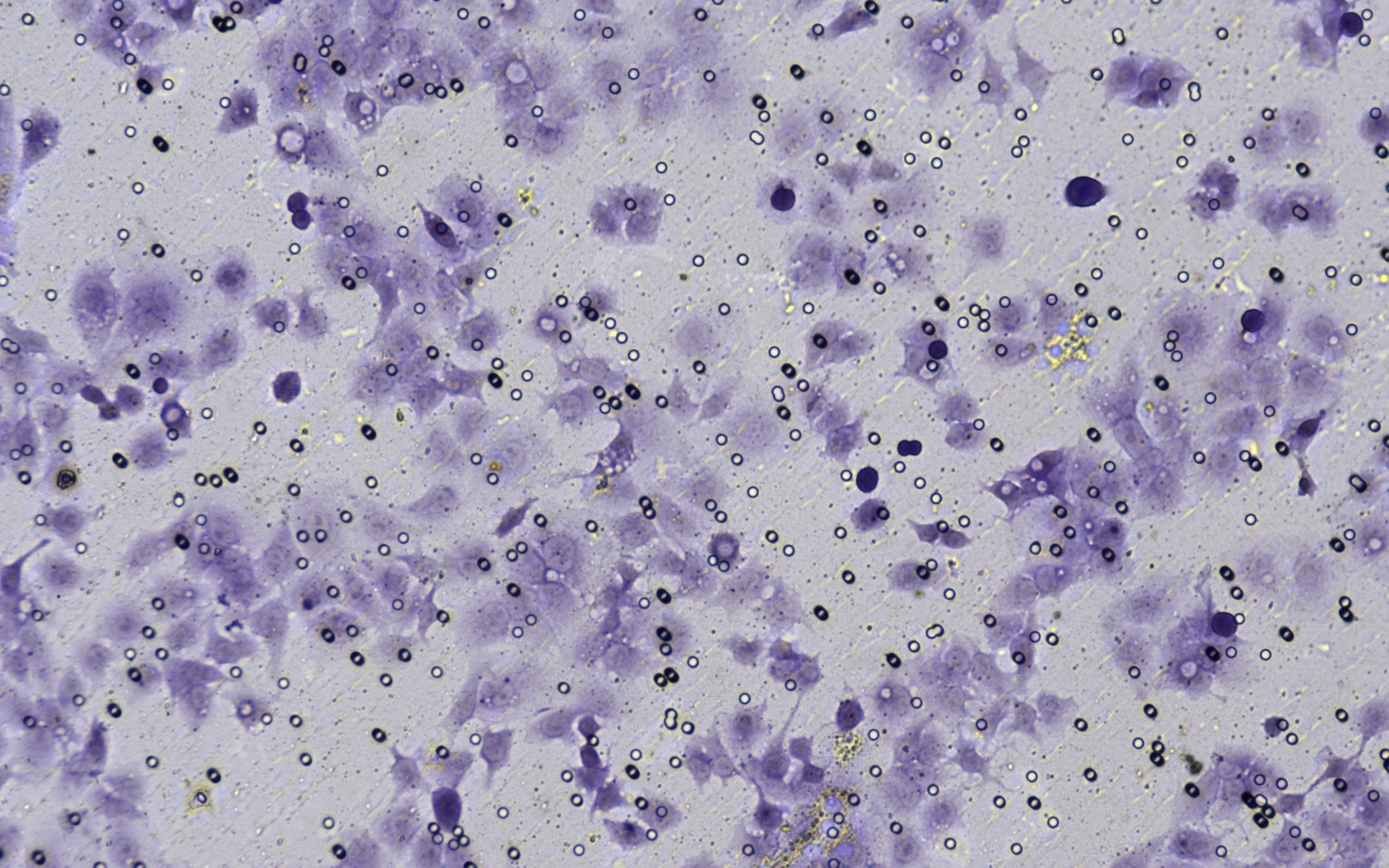

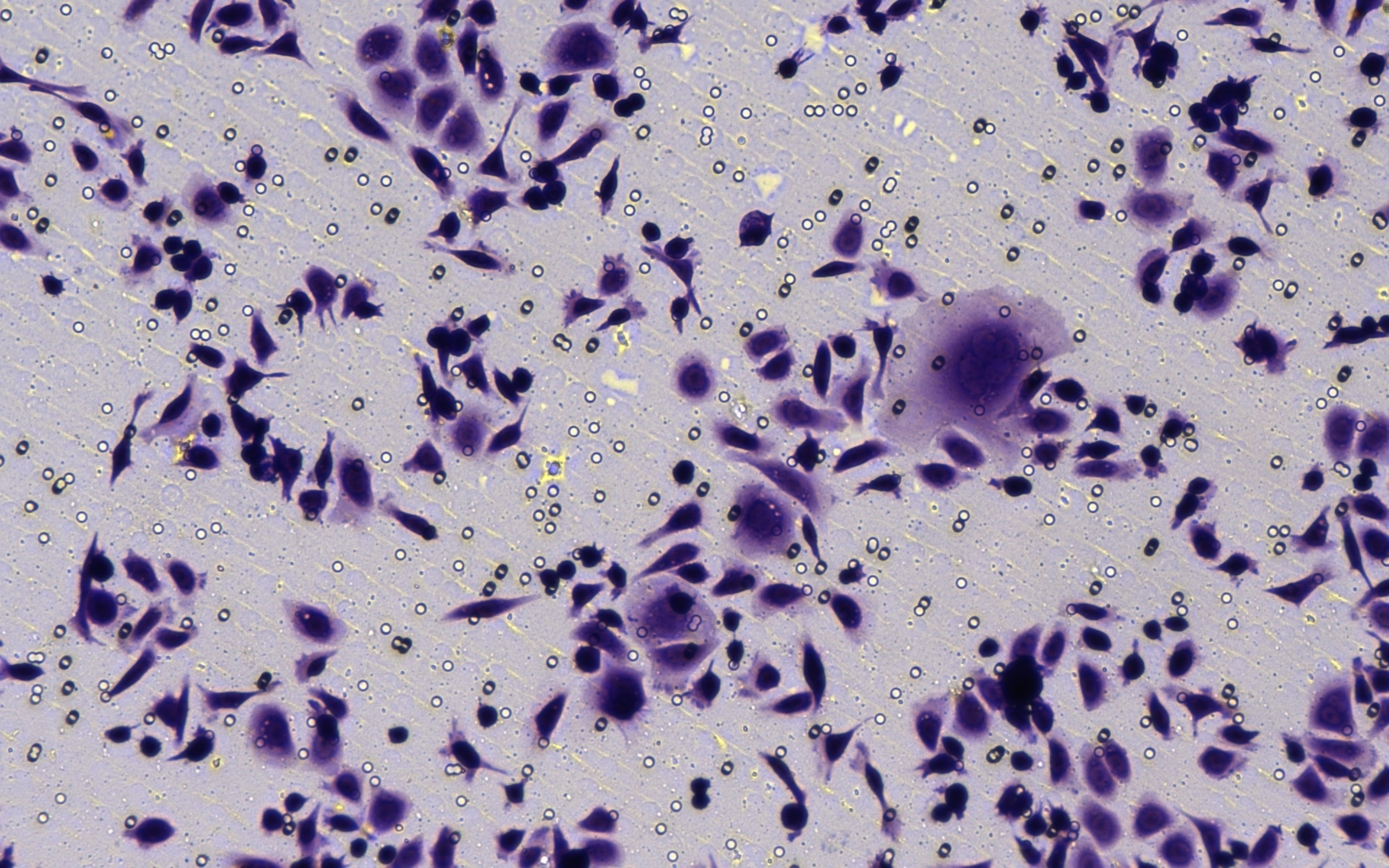

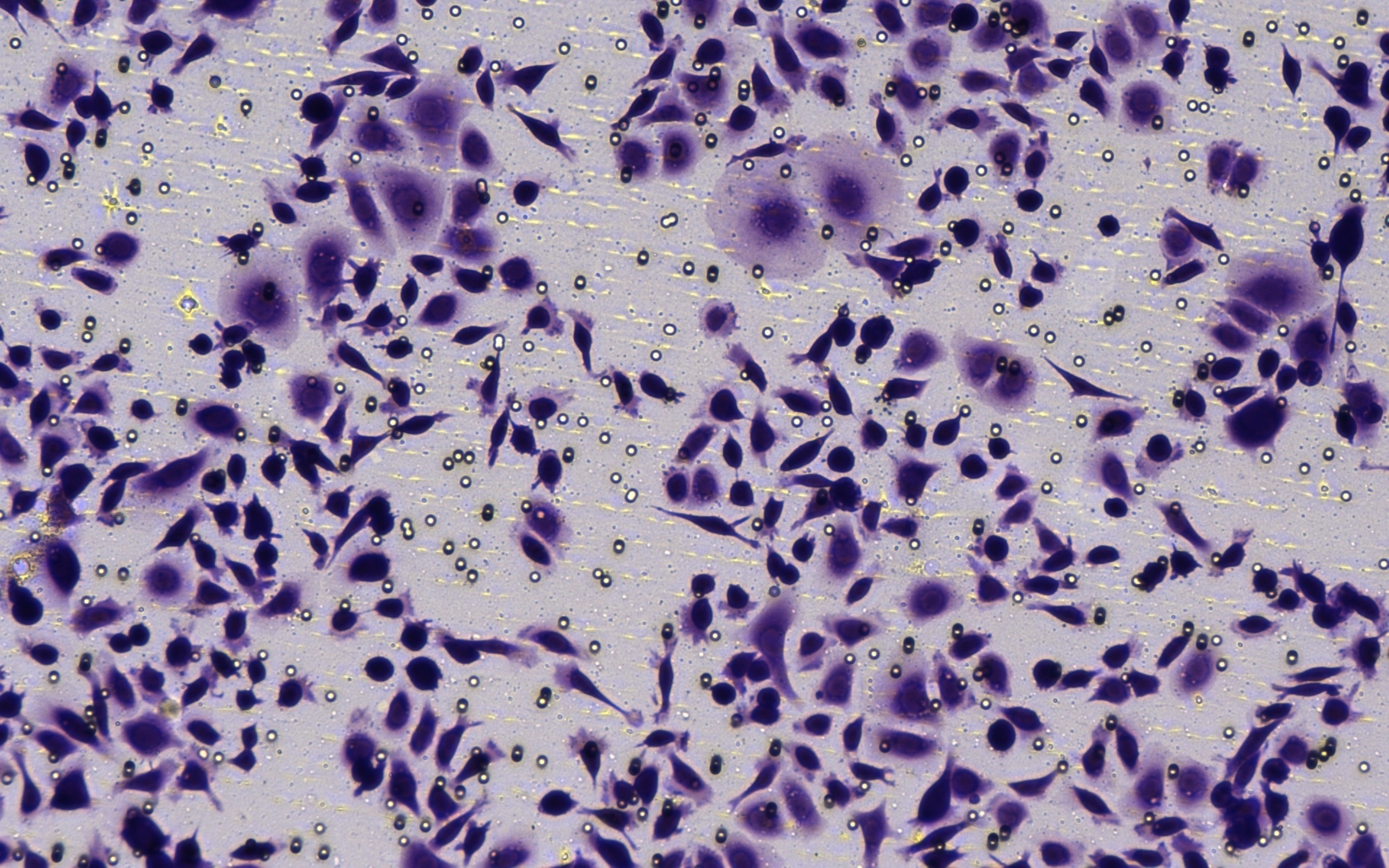

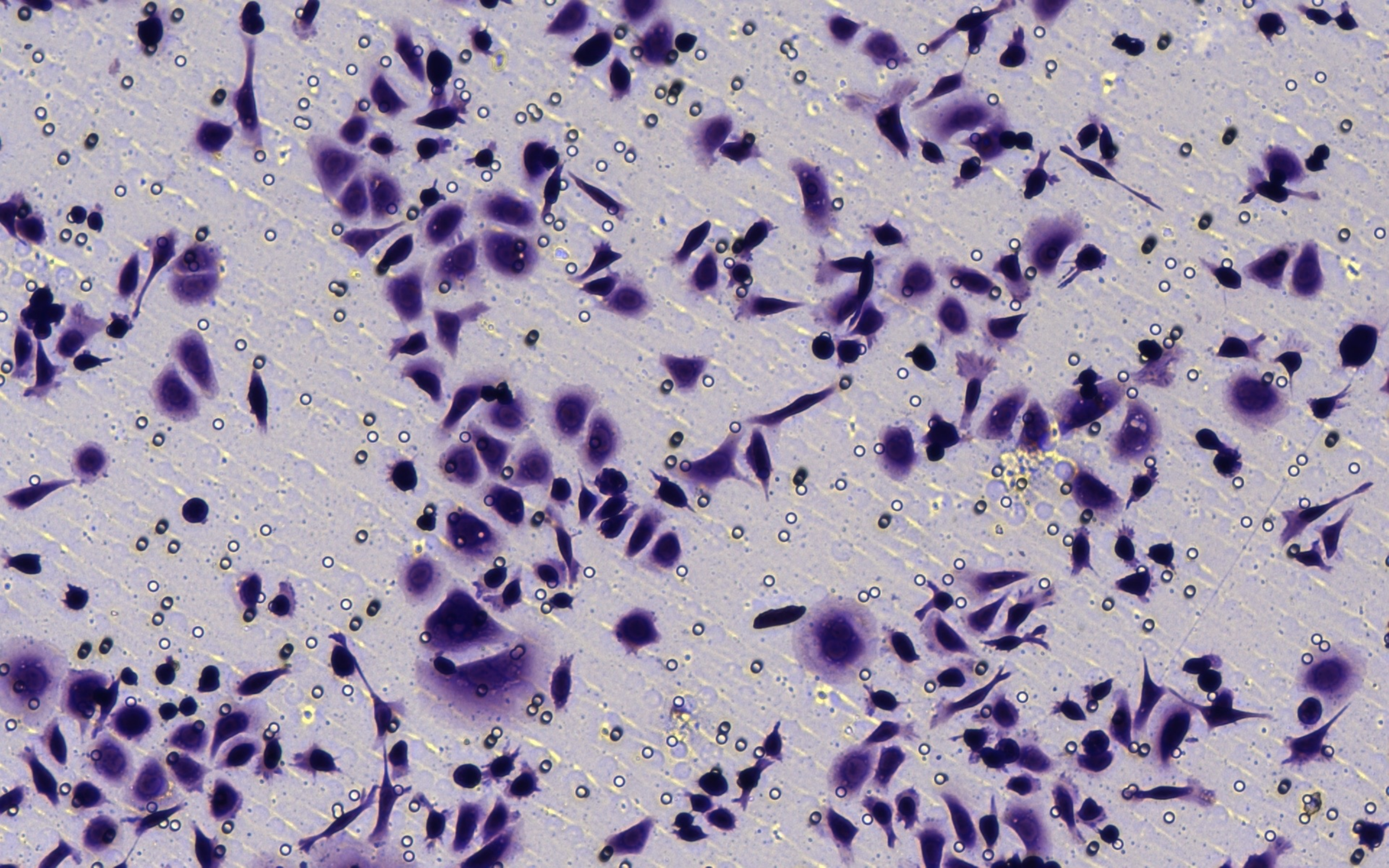

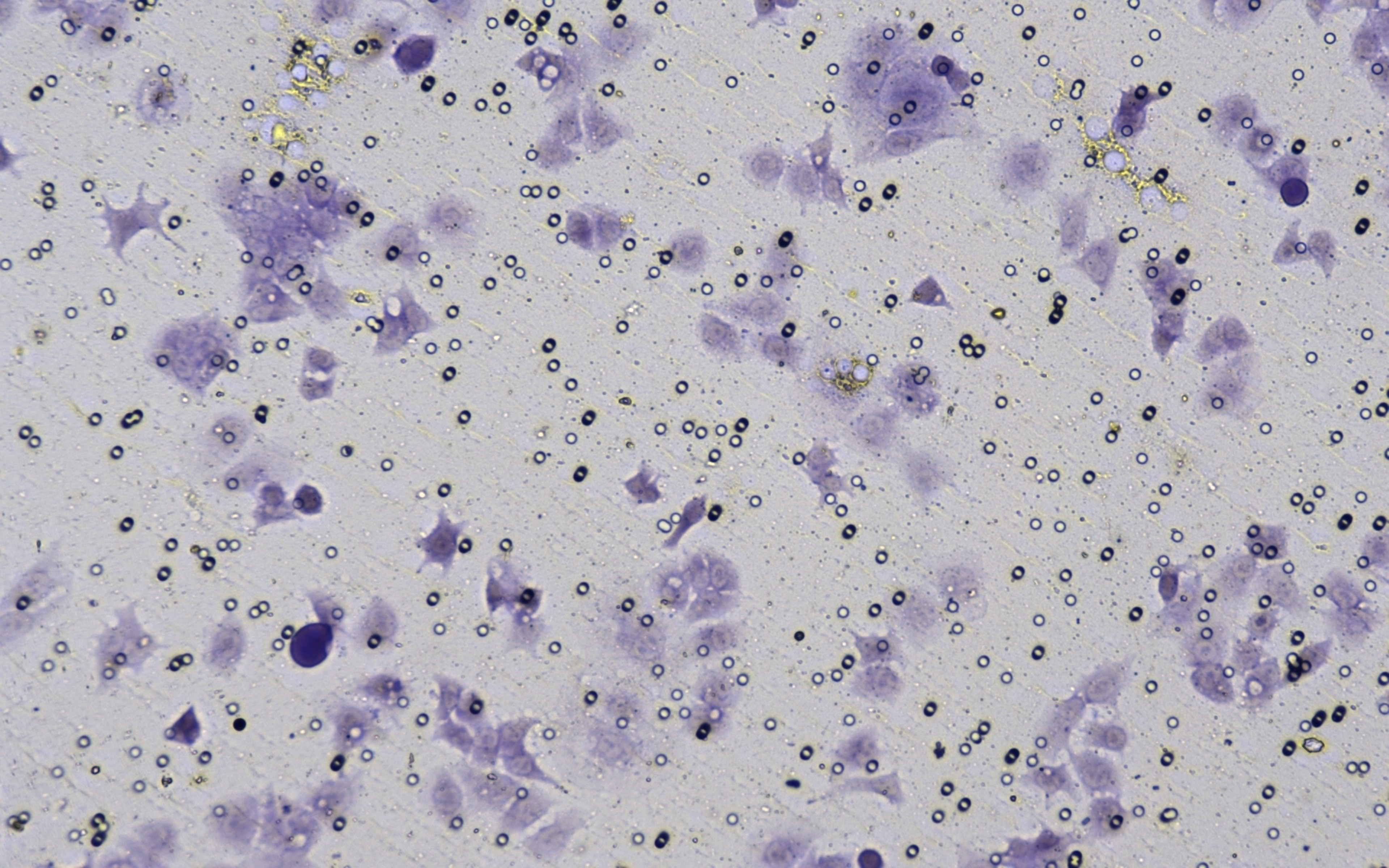

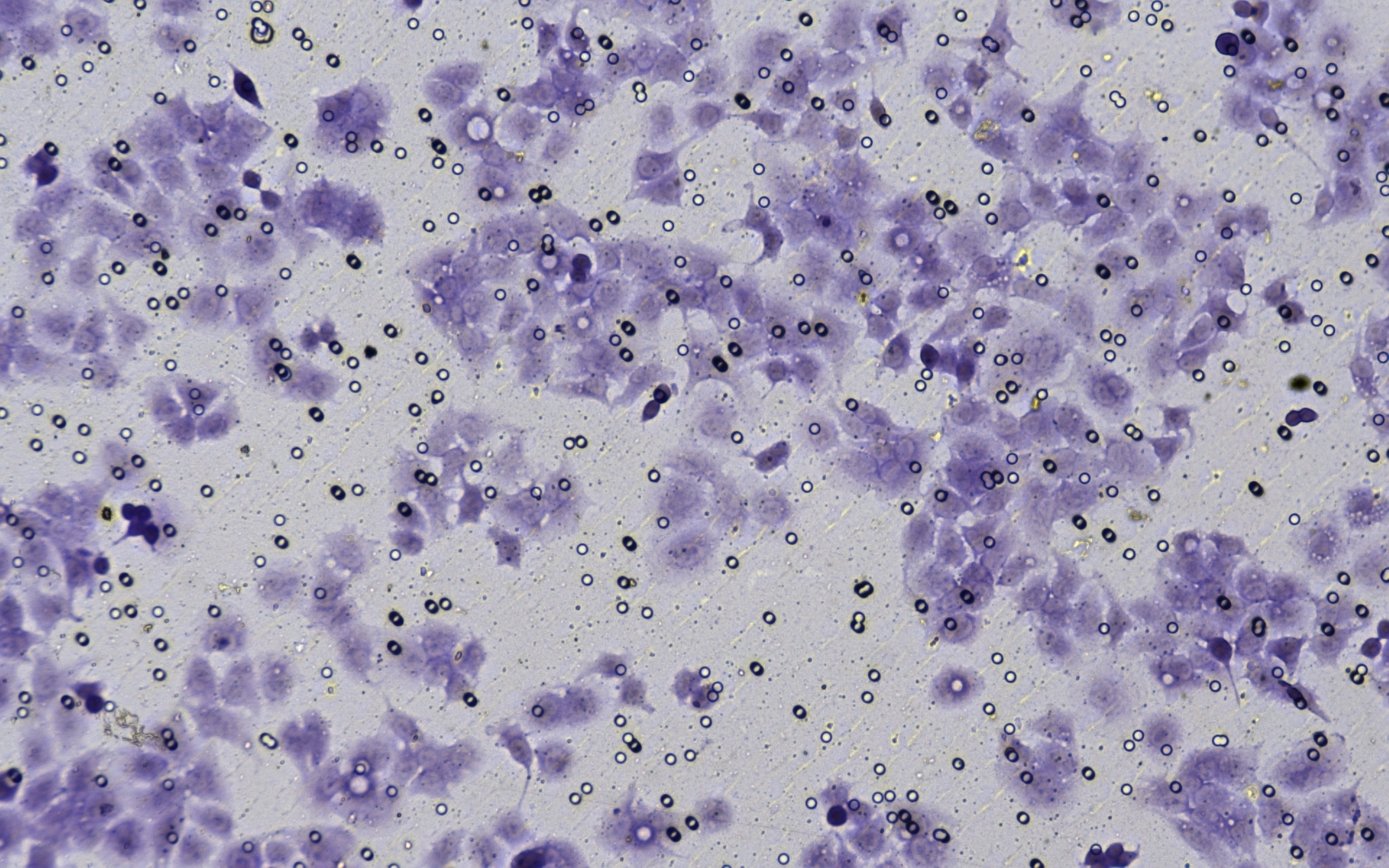

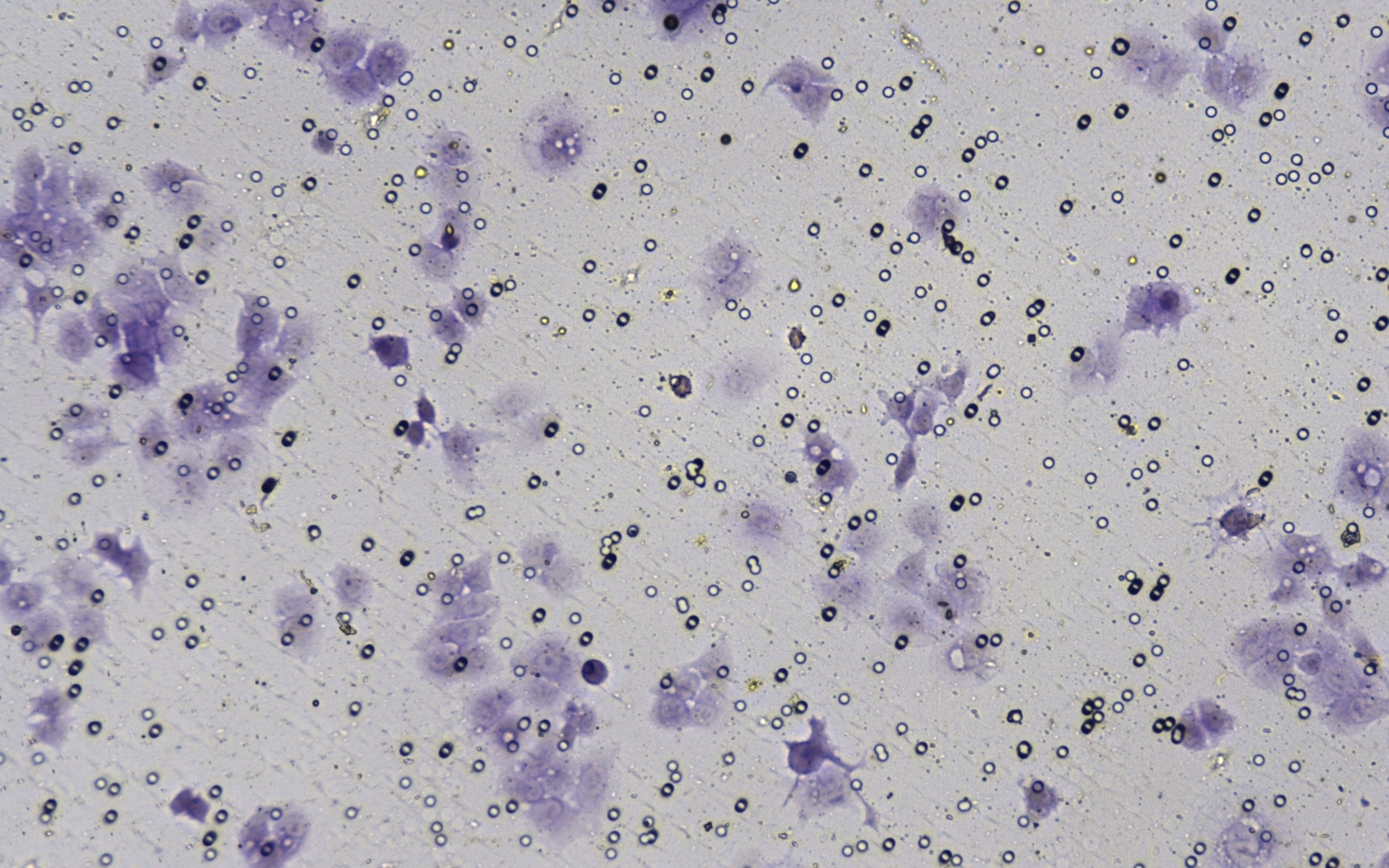

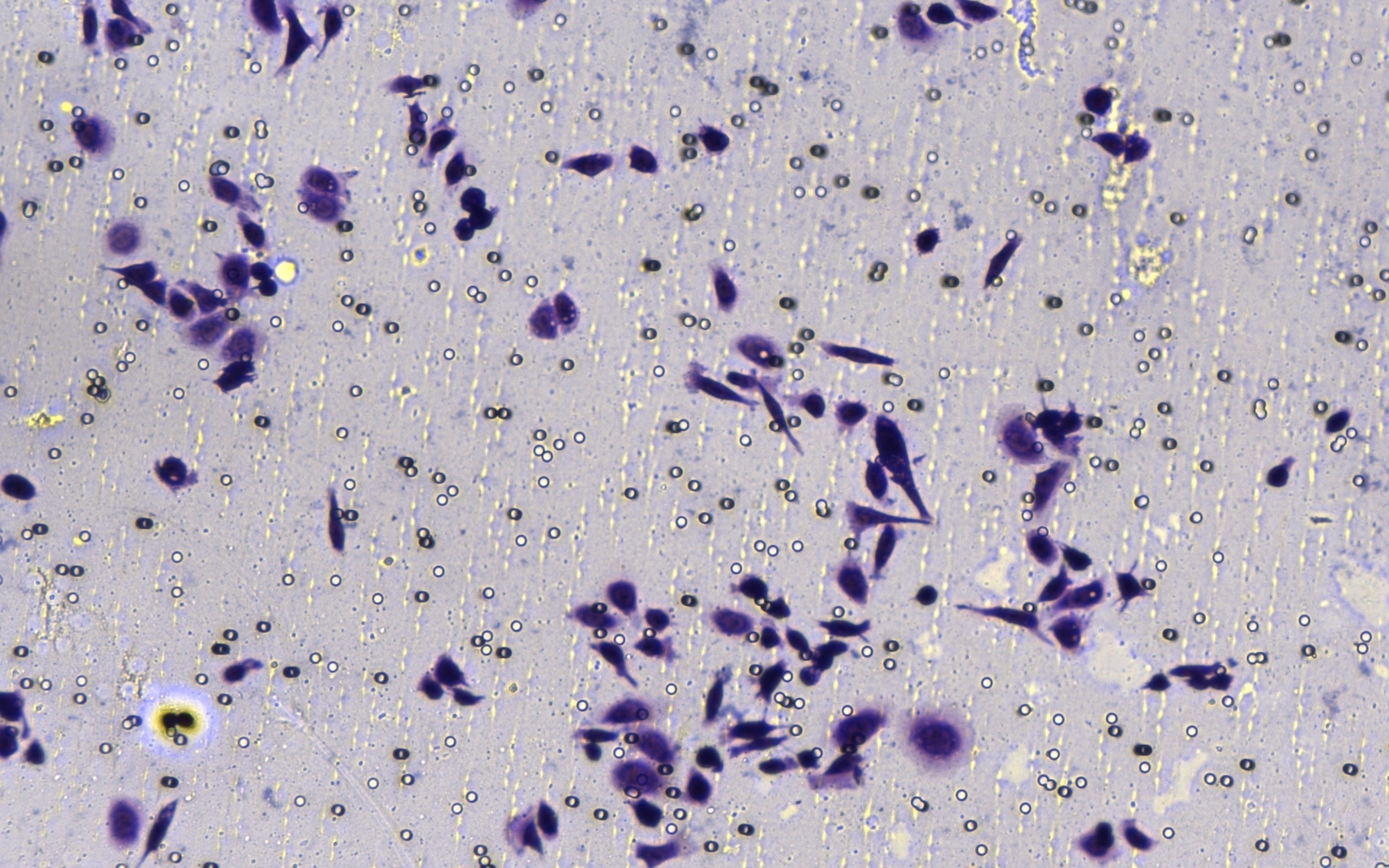

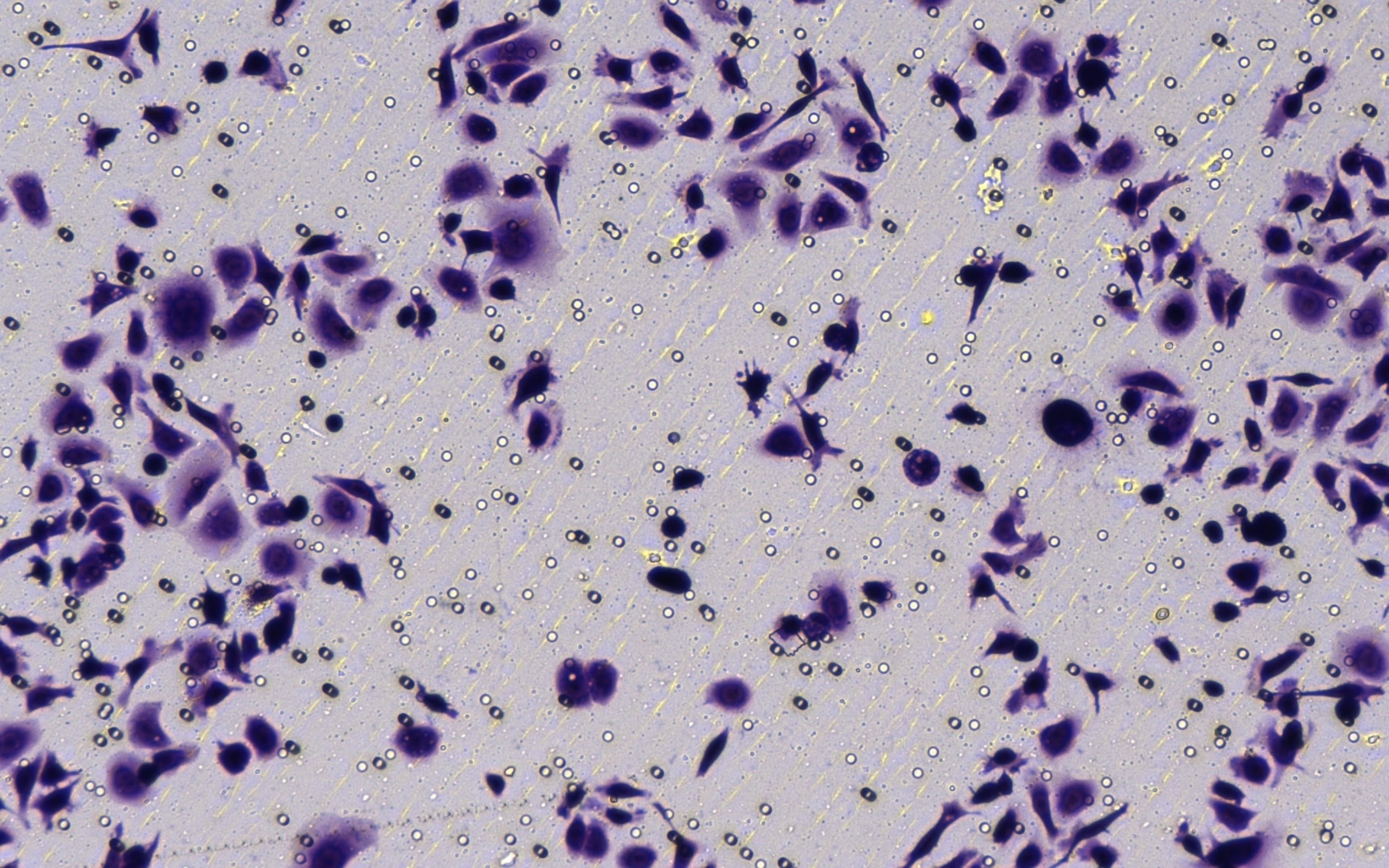

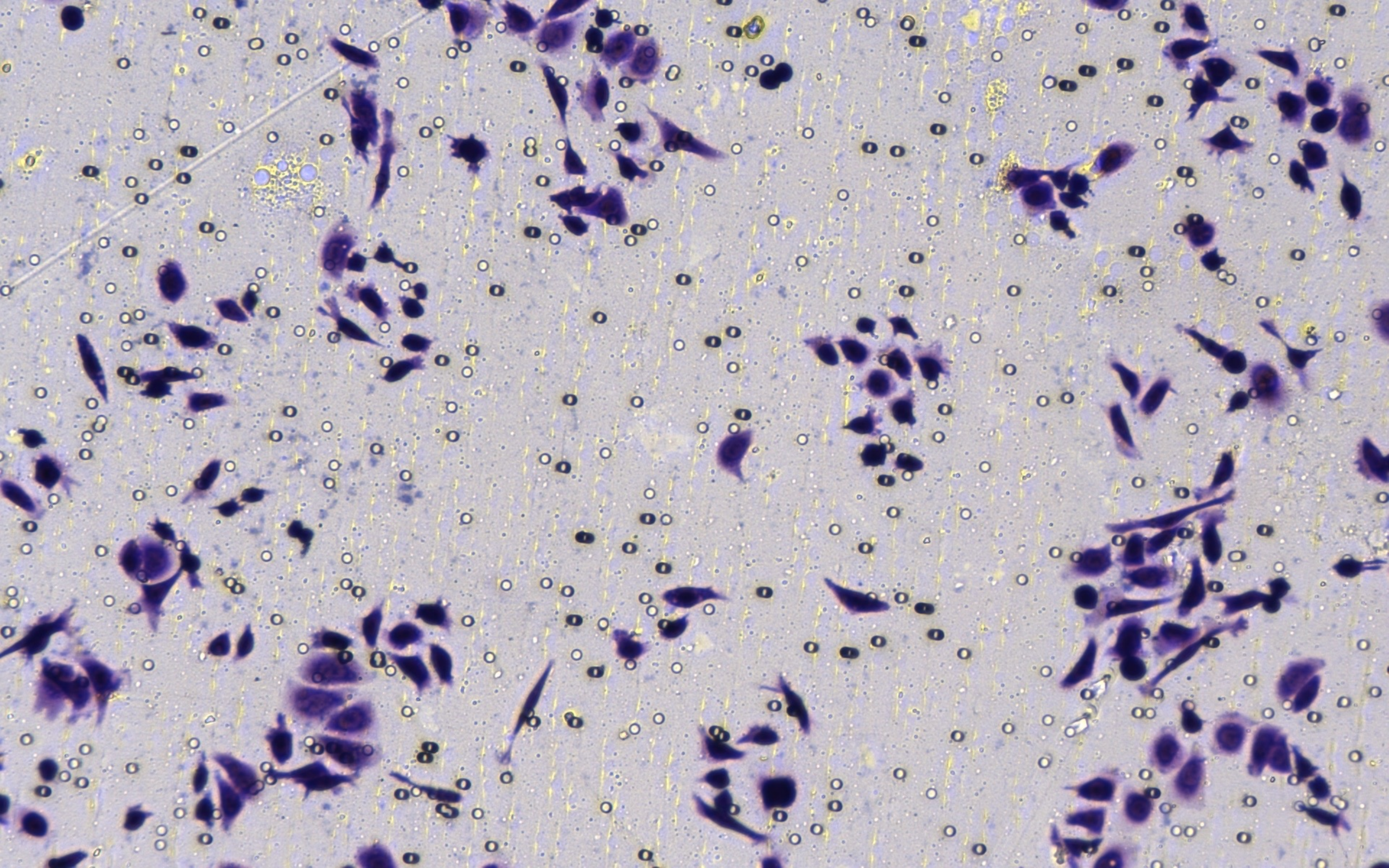

Supplement: Supplementary file 4 [file DataSheet_4.pdf]

**Figure 5c**

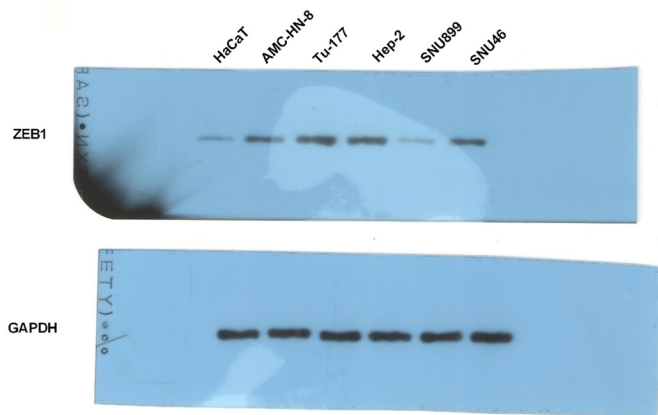

**Figure 5d**

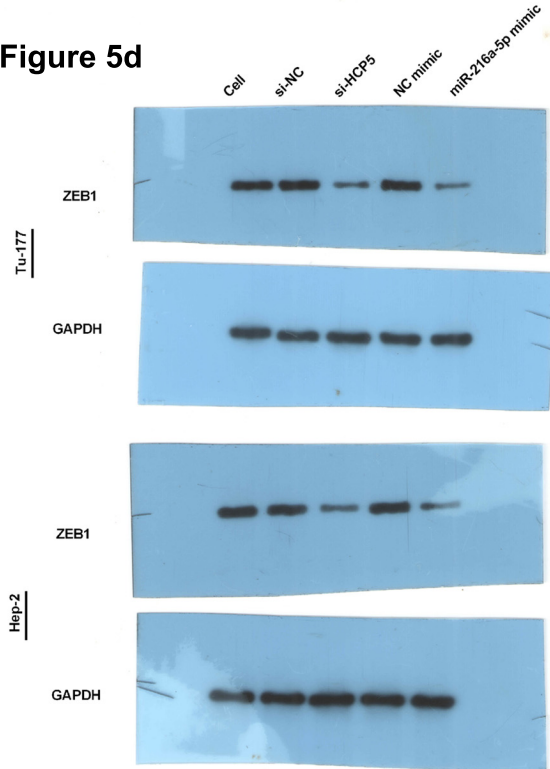

**Figure 6b**

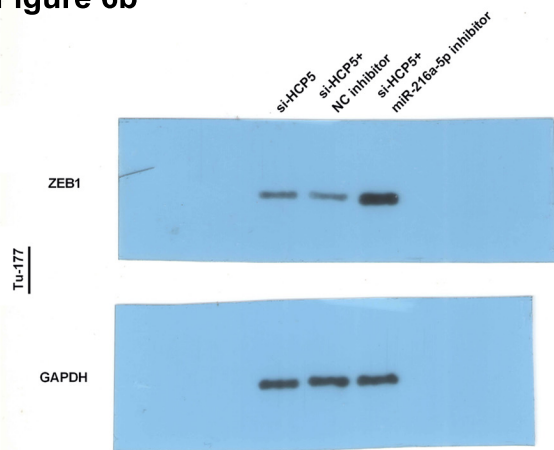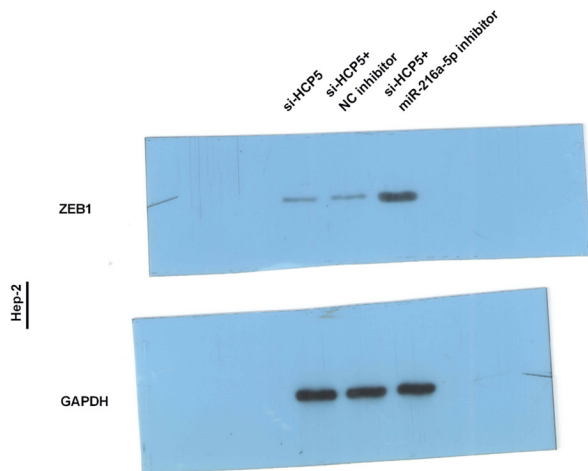

Supplement: Supplementary file 5 [file DataSheet_5.pdf]
